# The impact of metamorphosis and size on the aquatic feeding kinematics of the Iberian ribbed newt (*Pleurodeles waltl* Michahelles, 1830)

**DOI:** 10.1242/bio.062752

**Published:** 2026-09-28

**Authors:** Isabelle Toussaint-Lardé, Merlin Hugon-Daune-Fäs, Anthony Herrel, Anne-Claire Fabre

**Affiliations:** ^1^ Département Adaptations du Vivant, Mécanismes Adaptatifs et Evolution, UMR 7179, Muséum national d'Histoire naturelle CNRS, 75005 Paris, France; ^2^ Department of Biology, Institute of Ecology and Evolution, Universität Bern, 3012 Bern, Switzerland; ^3^ Abteilung Wirbeltiere, Naturhistorisches Museum Bern, 3005 Bern, Switzerland; ^4^ Department of Biology, Evolutionary Morphology of Vertebrates, Ghent University, 9000 Ghent, Belgium; ^5^ Department of Biology, University of Antwerp, 2610 Wilrijk, Belgium; ^6^ Department of Life Sciences, Natural History Museum, London SW7 5BD, UK

**Keywords:** Suction feeding, Ontogeny, Salamandridae, Unidirectional flow, Bidirectional flow

## Abstract

Metamorphosis involves abrupt morphological changes separating larval and adult stages. The adaptive decoupling hypothesis proposes that metamorphosis breaks the correlations among the phenotypes expressed at the different life stages, enabling their independent evolution. This should be reflected in differences in functional performance linked to the body parts remodelled during metamorphosis. Moreover, changes in body size throughout ontogeny can also influence functional performance. We investigated the influence of metamorphosis and size on suction feeding kinematics in larvae and metamorphs of Iberian ribbed newts using high-speed video analyses. Our results suggest that Iberian ribbed newts' ability to capture larger prey increases throughout ontogeny. Furthermore, apart from buccal expansion metrics, all feeding variables were significantly affected by the interaction between size and developmental stage. Notably, most duration variables increased with size in larvae, while decreasing with size in metamorphs. This pattern may reflect greater cranial ossification after metamorphosis, enabling more direct muscle force transmission and, consequently, faster movements relative to size. Finally, although requiring further investigation, our results suggest that larvae may enhance suction and prey retention through labial lobes and gill slits, whereas metamorphs may compensate for the loss of these larval structures through faster movements enabled by cranial ossification.

## Teaser

**Summary:** In the Iberian ribbed newt, although both larvae and adults use suction feeding to capture prey, metamorphosis modifies the relationship between body size and most of the feeding kinematic variables.

## INTRODUCTION

Metamorphosis is a developmental process characterized by drastic morphological changes transforming a larva into an adult (e.g. Laudet, 2011; Ten Brink, 2018; Ten Brink et al., 2019; Thompson, 2021). It is a metabolically costly process, as it involves an anatomical reorganization of the body plan (e.g. Ten Brink et al., 2019; Padilla et al., 2024). Because of this extensive remodelling, individuals may even experience difficulties feeding (Deban and Marks, 2002), often lose body mass, and are more vulnerable to predators (Wassersug and Sperry, 1977; Geffen et al., 2007; Ten Brink et al., 2019). Yet metamorphosis remains a prevalent ontogenetic process in the animal kingdom, occurring in over 80% of insects and 50% of vertebrates (Werner, 1988; Laudet, 2011; Belles, 2023; Manthey et al., 2024). This prevalence suggests that the benefits of metamorphosis may outweigh its costs.

Indeed, the existence of distinct life stages may enhance the ability of individuals to cope with the different selective pressures encountered throughout ontogeny. Some of these pressures are shared between larval and adult stages (e.g. resource acquisition and predator avoidance), whereas others are stage specific (e.g. adults experience additional pressures related to mate finding, reproduction, and dispersal). In this context, the acquisition of a new phenotype through metamorphosis may, on the one hand, allow post-metamorphic individuals to rely on different food resources compared with pre-metamorphic individuals (Ten Brink et al., 2019). On the other hand, it may result in each life stage having a phenotype better suited to its specific ecological and physiological demands (Istock, 1967; Moran, 1994; Ten Brink et al., 2019). This is summarized by the ‘adaptive’ (or ontogenetic) decoupling hypothesis, which proposes that metamorphosis breaks the correlations among the phenotypic traits related to fitness that are expressed at different life stages, thereby allowing post-metamorphic stages to evolve independently from pre-metamorphic ones and reducing inter-stage competition (Istock, 1967; Werner, 1988; Ebenman, 1992; Moran, 1994; Ten Brink et al., 2019).

Since the most apparent changes during metamorphosis are shifts in morphology, the ontogenetic decoupling has mostly been tested from a morphological perspective. However, if this hypothesis holds, it should also be reflected through differences in the performance of functions linked to body parts that are remodelled during metamorphosis. In vertebrates, the functional consequences of metamorphosis have mainly been studied for locomotion and feeding in salamanders, frogs, and fishes (Lauder and Shaffer, 1986; Reilly and Lauder, 1988; Shaffer and Lauder, 1988; Shaffer et al., 1991; Williams and Brown, 1992; Ashley-Ross, 1994; Reilly, 1996; Watkins, 2001; Azizi and Landberg, 2002; Deban and Marks, 2002; Wilson, 2005; Koumoundouros et al., 2009; Johansson et al., 2010). Several studies have reported marked locomotor changes after metamorphosis. For instance, Koumoundouros et al. (2009) reported that early metamorphosing stages of the gilthead seabream (*Sparus aurata*) exhibited significantly higher critical swimming speeds than later stages. Shaffer et al. (1991) further showed that burst speed and endurance were decoupled across life stages in the California tiger salamander (*Ambystoma californiense*). Lastly, Wilson (2005) reported reduced swimming but improved running performance following metamorphosis in the great crested newt (*Triturus cristatus*).

In contrast, other studies have found partial or no evidence of an impact of metamorphosis on locomotor performance. Ashley-Ross (1994), for example, reported no major changes in the limb kinematics of metamorphosed coastal giant salamander (*Dicamptodon tenebrosus*). Likewise, Williams and Brown (1992) and Azizi and Landberg (2002) found no post-metamorphic changes in escape performance in the winter flounder (*Pleuronectes americanus*) and in the northern two-lined salamander (*Eurycea bislineata*), respectively. Watkins (2001) found that larval swimming and juvenile jumping performance were correlated in the Pacific tree frog (*Pseudacris regilla*). On the contrary, Johansson et al. (2010) found a decoupling between larval swimming and juvenile jumping performance in the common frog (*Rana temporaria*), but this decoupling seemed only partial, as jumping performance was linked to juvenile body size, which was in turn determined by their size at the end of the tadpole stage.

As for studies concerning feeding performance, they have likewise yielded contrasting results. Reilly and Lauder (1988) and Lauder and Shaffer (1986) observed a significant decline in aquatic prey capture success following metamorphosis in the eastern newt (*Notophthalmus viridescens*) and the tiger salamander (*Ambystoma tigrinum*), respectively. Lauder and Shaffer (1986) also reported that adult *A. tigrinum* generated weaker suction pressures than larvae due to the resorption of gill slits at metamorphosis. However, Shaffer and Lauder (1988) found no effect of metamorphosis on aquatic feeding kinematics of *A. tigrinum*. Lastly, studies examining the transition from suction feeding to tongue prehension have also led to differing evolutionary interpretations. Deban and Marks (2002) argued that suction feeding and tongue prehension represent conflicting functional demands in plethodontid salamanders, whereas Reilly (1996) suggested that tongue prehension evolved through a gradual transformation of ancestral suction feeding mechanisms. Together, these findings indicate that the functional consequences of metamorphosis may be species and trait dependent and can lead to divergent evolutionary interpretations.

In addition to metamorphosis, changes in body size occurring throughout ontogeny are also expected to produce adjustments in the structure and consequently the function of the organism (Schmidt-Nielsen, 1984; Brown et al., 2000). For instance, regarding the feeding function of vertebrates, most studies have reported a positive correlation between feeding movement durations and body size (e.g. O'Reilly et al., 1993; Richard and Wainwright, 1995; Ferry-Graham, 1998; Wainwright and Shaw, 1999; Hernández, 2000; Robinson and Motta, 2002).

The present study quantifies the feeding kinematics in an ontogenetic series of fully aquatic Iberian ribbed newts (*Pleurodeles waltl* Michahelles, 1830). Interestingly, contrary to most biphasic species, in which aquatic larvae metamorphose into terrestrial forms, Iberian ribbed newts maintain a fully aquatic lifestyle when living in permanent water bodies (e.g. García-Paris, 1985; Raffaëlli, 2007; Sparreboom, 2014; Salvador, 2014; Joven et al., 2015). Consequently, aquatic metamorphs have to cope with the same environmental constraints as larvae. Moreover, rather than shifting to a terrestrial feeding mode such as tongue or jaw prehension, they continue to rely on suction feeding like the larvae. This strategy involves expanding the buccal cavity, which generates a wave that sucks water and prey into the mouth (Fig. 1, Movies 1 and 2; Deban, 2003; Heiss et al., 2013, 2015; Lukanov et al., 2016; Toussaint-Lardé et al., 2025). As a result, it remains unclear whether suction feeding kinematics differ between larvae and metamorphs and whether such differences are driven by changes in body size and/or by metamorphosis.

In line with the adaptive decoupling hypothesis, we expect the feeding kinematics to be influenced by developmental stage. Indeed, the cranium is remodelled during metamorphosis (Heiss and Grell, 2019; Smirnov et al., 2020); moreover, gills are resorbed, and gill slits are closed, which impacts how water is expelled from the oral cavity following prey capture (Lauder and Shaffer, 1986; Heiss and Grell, 2019). Whereas larvae use a unidirectional flow system, expelling water at the back of the head through the gill slits (Fig. 1A; Lauder and Shaffer, 1986; Heiss and Grell, 2019), metamorphs must rely on a bidirectional flow system in which water flow is reversed and exits through the mouth (Fig. 1B; Lauder and Shaffer, 1986; Heiss and Grell, 2019). In addition, we also expect changes in body size to affect the feeding kinematics, as metamorphs are usually bigger in size than larvae.

To summarize, we investigate how metamorphosis and body size influence suction feeding kinematics in Iberian ribbed newts (*P. waltl* Michahelles, 1830). To do so, we use larvae and metamorphs of varying sizes and test whether feeding kinematic variables are influenced by (1) body size, (2) developmental stage, or (3) a joint effect of both factors. If, as expected, both metamorphosis and body size affect the feeding kinematics, this should be reflected in the kinematic variables scaling differently with body size across developmental stages.

## RESULTS

The feeding kinematic variables described below are defined in detail in the Materials and Methods.

**Fig. 1.**
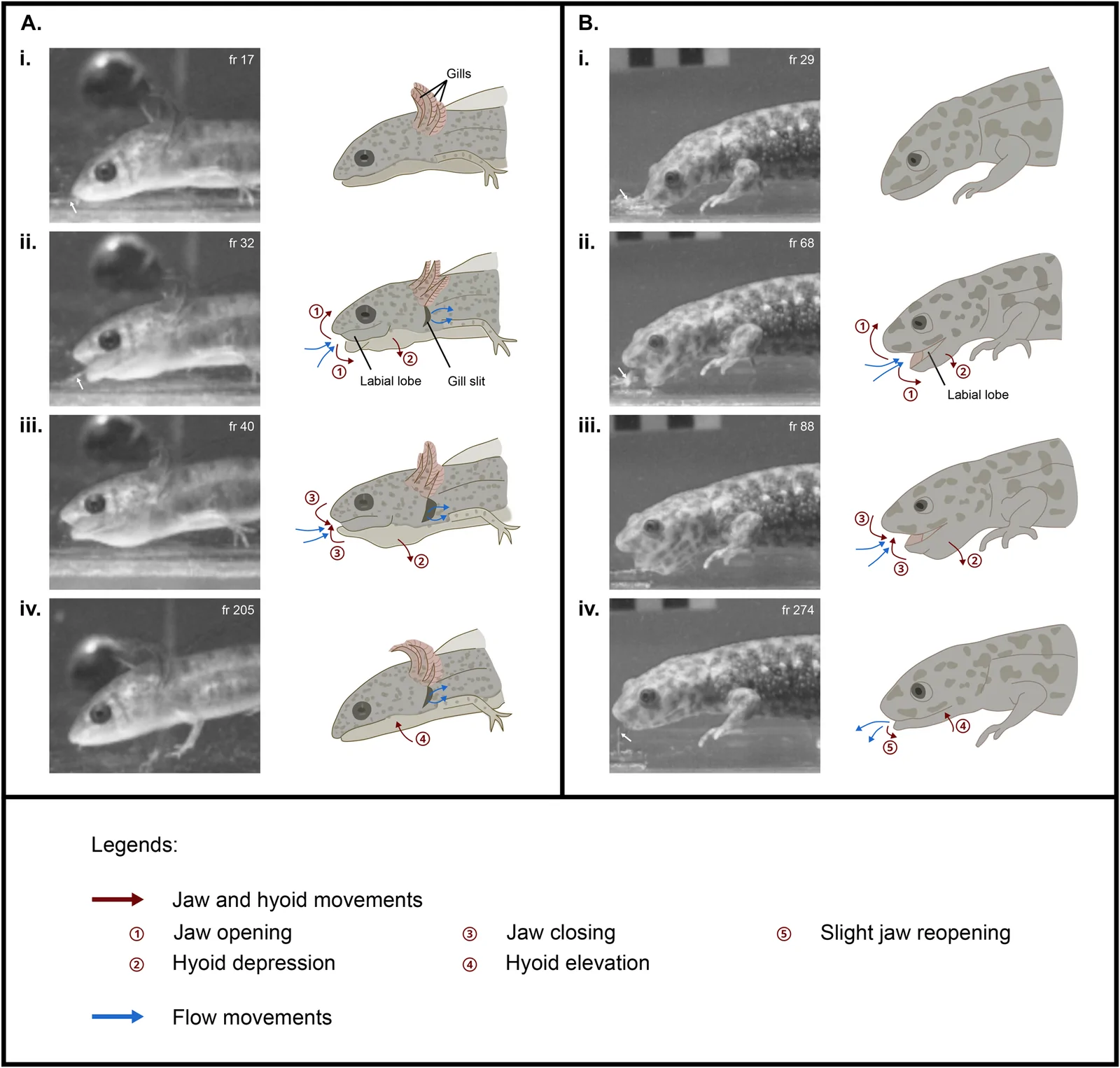
**Unidirectional versus bidirectional suction flow.** (A) Larval unidirectional flow system. (B) Metamorphic bidirectional flow system. Each panel shows representative video frames (fr; left) extracted from Movies 1 and 2 alongside a schematic interpretation of the observed movements (right). Before the strike, individuals usually position their snout close to the prey (A.i, B.i). In both stages, suction begins with rapid mouth opening followed almost simultaneously by hyoid depression (A.ii, B.ii). As the mouth starts to close after reaching maximum gape, the hyoid continues to depress until peak depression is reached (A.iii, B.iii). Finally, hyoid elevation compresses the buccal cavity and expels water (A.iv, B.iv). Notice that in larvae, early opening of the gill slits allows continuous expulsion of water at the back of the head (A.ii–A.iv). In metamorphs, where gill slits are resorbed, water flow is reversed and expelled through the slightly reopened mouth (B.iv). White arrows indicate the position of the prey. In the metamorph sequence, the reversal of water flow at the end of the strike pulls the prey forward while it is held between the jaws, causing it to protrude from either side of the mouth (B.iv).

### Results of the type II analyses of variance (ANOVAs)

The interaction between size and developmental stage significantly impacts all the angle and duration variables but has no effect on the buccal expansion metrics [maximum gape distance (MG) and maximum hyoid depression (Mhd); Table 1]. For MG, size is the only significant predictor, regardless of whether the data are fitted to the full model (including the interaction term) or the reduced model (without the interaction term; Table 1). For Mhd, both size and developmental stage have significant effects in the full model, whereas only developmental stage remains significant in the reduced model (Table 1).

**Table 1.** Results of the type II ANOVAs

Kinematic variables Full model (with the interaction term)	Developmental stage		Size		Developmental stage×size	
	*F*	*P*	*F*	*P*	*F*	*P*
MGA	6.832	0.017*	2.042	0.173	6.492	0.020*
MG (AIC=−201.9; BIC=−181.1)	2.209	0.153	22.096	<0.001*	1.813	0.194
TMG	38.905	<0.001*	12.823	0.003*	36.859	<0.001*
JCD	32.631	<0.001*	1.108	0.308	33.604	<0.001*
DG	36.335	<0.001*	3.952	0.064	37.723	<0.001*
MHA	6.979	0.014*	0.414	0.533	7.559	0.013*
Mhd (AIC=−311.9; BIC=−291.2)	6.385	0.017*	5.943	0.023*	4.193	0.052
TMhd	25.865	<0.001*	4.978	0.042*	25.795	<0.001*
DhdE	26.823	<0.001*	1.119	0.305	23.849	<0.001*
PCD	24.435	<0.001*	0.221	0.644	21.658	<0.001*
Kinematic variables Reduced model (without the interaction term)	Developmental stage		Size			
	*F*	*P*	*F*	*P*		
MG (AIC=−202.3; BIC=−184.6)	0.389	0.543	18.288	<0.001*		
Mhd (AIC=−310.5; BIC=−292.7)	4.554	0.037*	2.007	0.162		

For MG and Mhd, both a full model (including the interaction term) and a reduced model (excluding the interaction term) were fitted because the interaction term was not significant. Model performance was compared using the AIC and the BIC; the model with the lower AIC and BIC values was considered to provide a better fit to the data. Definitions of the feeding kinematics are provided in the Materials and Methods section. Raw data were transformed using the decadic logarithm (‘log10’ transformation), except for the MHA analysis, for which the natural logarithm of 1+*x* (‘log1p’ transformation) was used. * denotes a significant impact of the factor.

### Predicted linear regressions (Figs 2, 3, 4 and 5)

For duration and angle variables, separate linear regressions were fitted for each developmental stage because of the significant interaction between size and developmental stage. In larvae, all durations are significantly and positively related to size (Figs 3A–C and 4A) except for the duration of hyoid elevation (DhdE; Fig. 4B) and prey capture duration (PCD; Fig. 4C), which show positive but non-significant relationships with size. In metamorphs, all durations are significantly and negatively related to size (Figs 3 and 4). The maximum gape angle (MGA) and maximum head angle (MHA) show positive but non-significant relationships with size in larvae (Figs 2B and 5). In metamorphs, however, MGA shows a significant positive relationship with size (Fig. 2B), whereas MHA shows a significant negative relationship with size (Fig. 5).

**Fig. 2.**
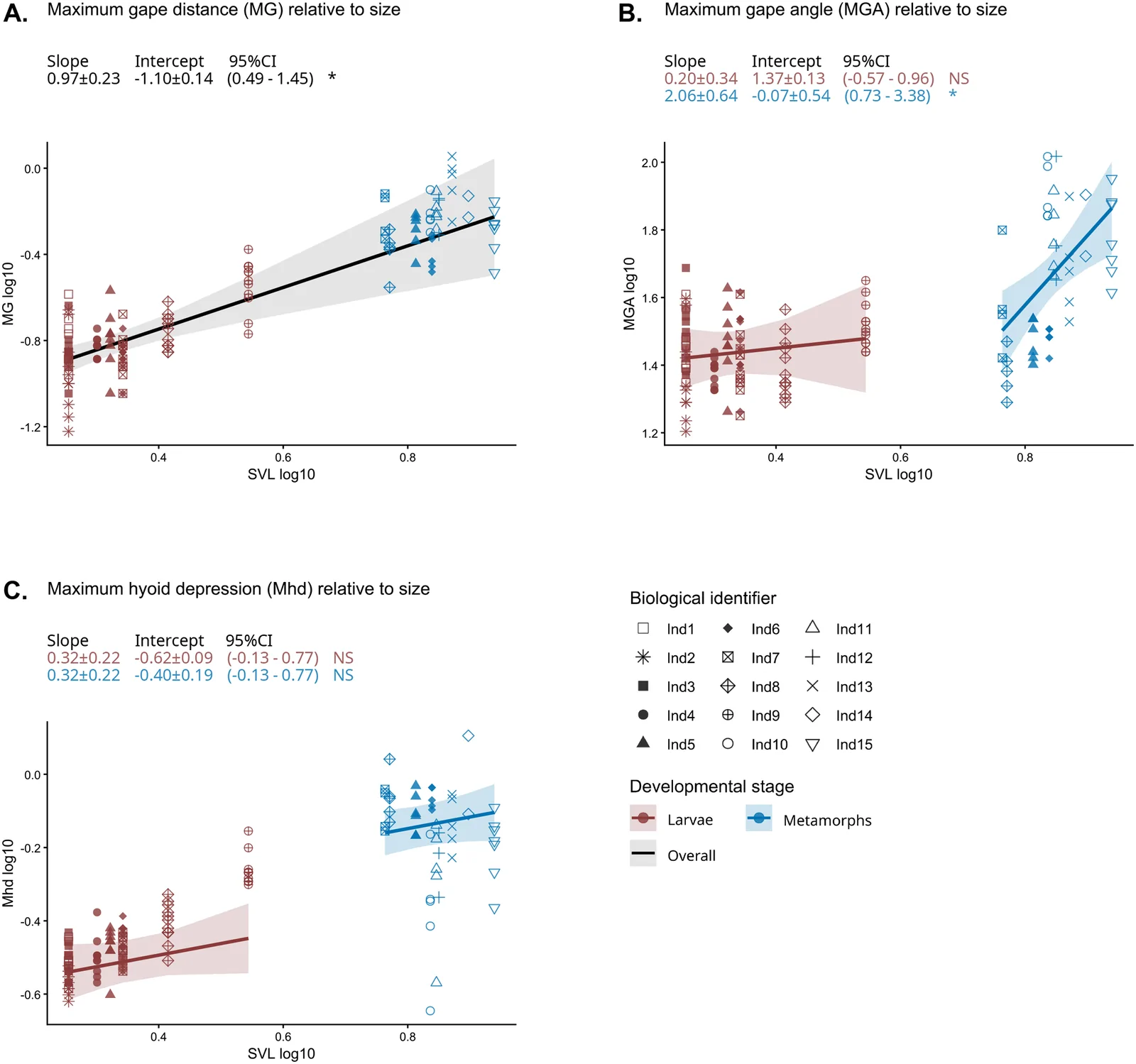
**Amplitude of buccal expansion relative to size (SVL).** (A) MG relative to size. (B) MGA relative to size. (C) Mhd relative to size. Graphs show both the raw data and the predictions from the linear mixed-effects models. Regression lines and their shaded 95% CIs were generated using the ‘ggpredict’ function. Slope estimates (±standard error), intercepts (±standard error), and 95% CIs of the slopes were obtained using the ‘emtrends’ and ‘emmeans’ functions with the Satterthwaite approximation (package emmeans). The number of regression lines reflects the results of the type II analyses of covariance. For MG, only size had a significant effect; therefore, a single regression line is presented. For MGA, the interaction between size and developmental stage was significant, indicating that the effect of size differed between larvae and metamorphs; consequently, separate regression lines are shown for each developmental stage. For Mhd, only developmental stage had a significant effect, also resulting in separate regression lines for each developmental stage. Raw data were transformed using the decadic logarithm (log 10). * denotes a slope significantly different from zero, while ‘NS’ means the slope is not significantly different from zero.

**Fig. 3.**
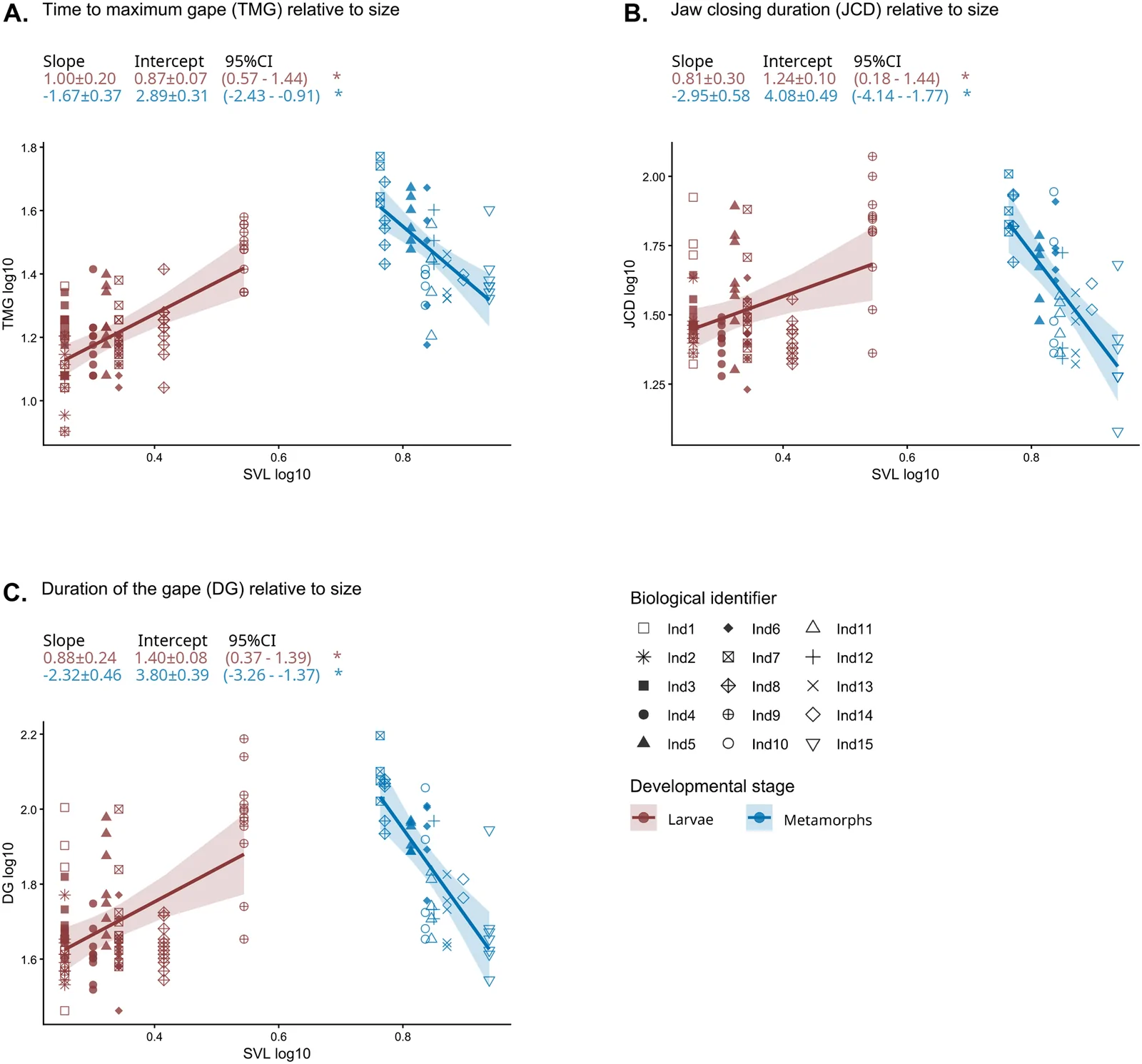
**Duration of mouth movements relative to size (SVL).** (A) TMG relative to size, (B) JCD relative to size, and (C) DG relative to size. Graphs show both the raw data and the predictions from the linear mixed-effects models. See Fig. 2 and Materials and Methods for details on how the regression lines were generated. Separate regression lines were fitted for each developmental stage because the interaction term in the ANOVAs was significant in all cases. Raw data were transformed using the decadic logarithm (log 10). * denotes a slope significantly different from zero (95% CI excludes zero), while ‘NS’ means the slope is not significantly different from zero (95% CI includes zero).

**Fig. 4.**
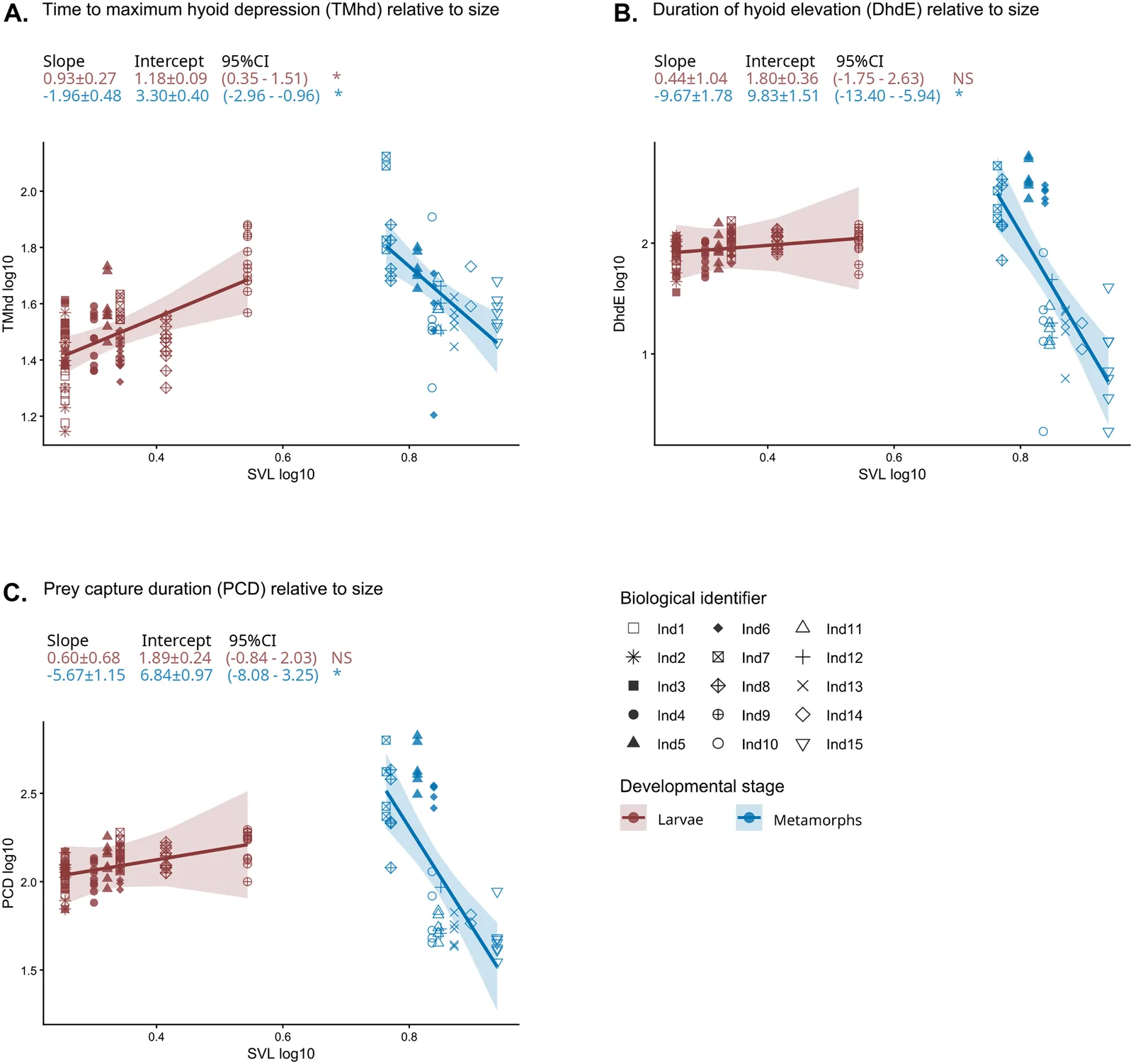
**Duration of hyoid movements and of total PCD relative to size (SVL).** (A) TMhd relative to size, (B) DhdE relative to size, and (C) PCD relative to size. See Fig. 2 and Materials and Methods for details on how the regression lines were generated. Separate regression lines were fitted for each developmental stage because the interaction term in the ANOVAs was significant in all cases. Raw data were transformed using the decadic logarithm (log 10). * denotes a slope significantly different from zero (95% CI excludes zero), while ‘NS’ means the slope is not significantly different from zero (95% CI includes zero).

**Fig. 5.**
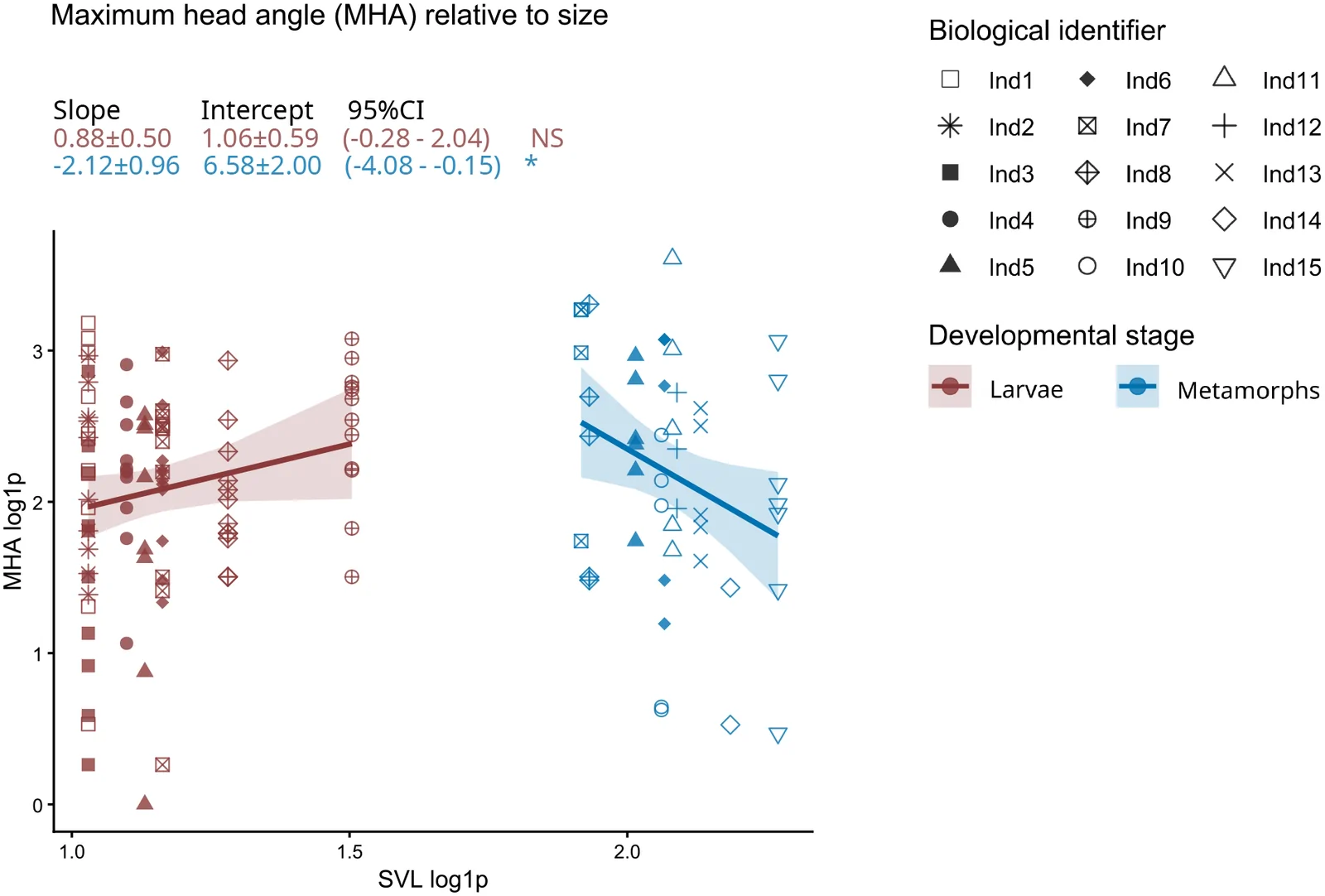
**MHA during suction feeding relative to size (SVL).** See Fig. 2 and Materials and Methods for details on how the regression lines were generated. Separate regression lines were fitted for each developmental stage because the interaction term in the ANOVAs was significant in all cases. Raw data were transformed using the natural logarithm of 1+*x* (‘log1p’ transformation) because the data contained one zero value. * denotes a slope significantly different from zero (95% CI excludes zero), while ‘NS’ means the slope is not significantly different from zero (95% CI includes zero).

For the buccal expansion metrics (MG and Mhd), the predicted regressions are based on the reduced models without the interaction term, which provided a better fit to the data [see Materials and Methods as well as the Akaike information criterion (AIC) and Bayesian information criterion (BIC) values in Table 1]. As MG is affected only by size, a single regression line was fitted across all developmental stages and shows a significant positive relationship with size (Fig. 2A). Separate regression lines were fitted for Mhd because it is affected only by developmental stage. Both the larval and metamorphic regression lines are positive but non-significant and differ only in their intercept, which is higher in metamorphs (Fig. 2C).

## DISCUSSION

### The amplitude of buccal expansion increases throughout ontogeny, likely expanding the range of prey sizes that Iberian ribbed newts can capture (Fig. 2)

During suction feeding, it is the buccal expansion – driven by mouth opening and hyoid depression – which generates the water flow that draws the prey into the predator's mouth (Fig. 1A.ii-iii and B.ii-iii; Deban, 2003; Heiss et al., 2013, 2015; China Holzman, 2014; Lukanov et al., 2016; Toussaint-Lardé et al., 2025). Our results show that MG increases with body size only (Fig. 2A). Although the lack of size overlap between larvae and metamorphs may limit the strength of this inference, the observed pattern suggests that individuals can capture larger prey as they grow. Interestingly, MGA shows a break of slope depending on the developmental stage, with a much steeper slope in metamorphs (0.20 in larvae versus 2.06 in metamorphs; Fig. 2B). This suggests that metamorphs open their mouth wider than predicted by body size alone. Indeed, if size alone constrained the maximum gape, the angle would have remained constant across all stages even if the MG increases with size. However, it is difficult to assess if this break of slope is due to a subtle change of mouth gape kinematics following metamorphosis or if it simply reflects the fact that metamorphs M4 and M6–M10 were fed bigger prey compared to other metamorphs (pieces of earthworm versus bloodworms). Consequently, we cannot completely exclude an effect of prey size on this variable. Nevertheless, any such effect appears modest, as no break of slope is observed for MG (Fig. 2A). Lastly, Mhd is mainly impacted by developmental stage, with metamorphs exhibiting a significantly greater Mhd, and consequently a larger buccal expansion volume, than larvae (intercept: −0.40 in metamorphs versus −0.62 in larvae; Fig. 2C), which may further enhance the ability of metamorphs to capture larger prey.

To summarize, although we cannot exclude a potential effect of the difference in prey size/type provided to some metamorphs on the buccal expansion variables, our results suggest that an increase in size likely enhances the ability to capture larger prey irrespective of developmental stage. This interpretation is consistent with the observations of Garcia-de-Lomas et al. (2012) and Escoriza et al. (2020), who reported that larger larvae consume larger prey, although neither study included metamorphs. More broadly, this pattern is widespread among suction feeding vertebrates (fishes: Werner, 1974; Keast, 1985; Wainwright and Richard, 1995; Wainwright et al., 2007; salamanders: Kusano et al., 1985; Whiteman et al., 1996; Toussaint-Lardé et al., 2025). However, our findings further suggest that metamorphosis reinforces this effect through a subtle shift in gape angle trajectory (unless this effect is due to the different meals provided to some metamorphs, which may be a confounding factor; Fig. 2B) and increased hyoid depression (Fig. 2C). Future studies investigating prey capture performance across a range of prey sizes and evaluating prey size preferences in larvae and metamorphs would provide a direct test of the interpretations proposed here.

### Metamorphs are faster than larvae, which may reflect greater cranial ossification as well as increased muscle mass

Interestingly, all duration variables increase significantly with size in larvae (except DhdE and PCD slopes, which are positive but not significant), whereas all durations decrease significantly with size in metamorphs (Figs 3 and 4). The absolute slope estimates of the duration variables are between 1.67 and 21.98 times greater in metamorphs than in larvae (Figs 3 and 4). This indicates that relative to their size, metamorphs have faster suction movements compared to larvae. Although the differences in prey size/type provided to some metamorphs cannot be completely ruled out as a potential source of variation, this explanation seems unlikely to explain the observed pattern, as larger prey would be expected to increase, rather than decrease, movement durations. These patterns may therefore be more likely linked to the remodelling of the skull that occurs during metamorphosis. Cranial ossification was not quantified in the present study, but previous work has described the underlying mechanism. Smirnov et al. (2020) showed that, at metamorphosis, there is a peak in thyroid hormone level, which not only induces the differentiation of the bones already ossified during the early larval stage into their metamorphic forms but also induces the ossification of new bones (see figure 1A,B in Smirnov et al., 2020). As a result, although cranium ossification is not quantified in our study, the metamorphic skull in salamanders is generally much more ossified and differentiated than the larval one, which remains largely cartilaginous (see figure 1A,B in Smirnov et al., 2020). Muscle movements are more effective when acting on ossified bones, as rigid structures allow direct transmission of muscular force due to the differences in mechanical properties between bone and cartilage (Currey, 2002). In contrast, cartilaginous bones are more flexible and tend to deform during contraction, dissipating part of the generated force and thereby reducing muscular efficiency (Currey, 2002). This may explain why, relative to size, metamorphs have faster movements compared to larvae (Figs 3 and 4). Moreover, muscular mass and cross-sectional area generally increase with size, and coordination improves with age (Herrel and Gibb, 2006; Carrier, 1996), which may further enhance movement rapidity in metamorphs compared to larvae. Lastly, the fact that metamorphs elevate their cranium to a lesser extent compared to larvae during suction feeding (MHA; Fig. 5) may also reflect improved coordination of head movements in metamorphs during prey capture.

### Suction efficiency may rely on different morphological features in larvae and metamorphs

Relative to body size, time to maximum gape (TMG) and time to maximum hyoid depression (TMhd) are reached faster in metamorphs than in larvae, with slopes that are 1.67 and 2.11 times steeper, respectively (Figs 3A and 4A). Consequently, peak flow velocity may be achieved earlier in metamorphs, resulting in an increase in suction force (Wainwright and Day, 2007).

However, this does not mean that larval suction is inefficient compared to that of metamorphs. Indeed, both larvae and metamorphs possess labial lobes, i.e. flaps of skin joining the upper and lower jaws (Deban, 2003; Toussaint-Lardé et al., 2025). As they occlude lateral portions of the mouth, labial lobes reduce mouth aperture and thereby increase water flow velocity during suction and direct the flow to the front of the mouth (Deban, 2003; Wainwright and Day, 2007; Van Wassenbergh and Heiss, 2016). However, labial lobes are much more developed in larvae, where they occlude the entire lateral margins of the mouth, whereas they are markedly reduced in metamorphs (Fig. 1; Fig. S1, Movies 1 and 2). Van Wassenbergh and Heiss (2016) demonstrated that in adult smooth newts (*Lissotriton vulgaris*) that develop labial lobes during the breeding season, suction force and distance are increased by 15%. Yet the labial lobes of breeding adult smooth newts, like those of metamorph Iberian ribbed newts, occlude only a small portion of the lateral part of the mouth (see figure 1b in Van Wassenbergh and Heiss, 2016). Therefore, in larvae, where labial lobes occlude the entire lateral margins of the mouth (Fig. S1), suction force and distance likely increase to a greater extent.

Moreover, larvae possess gill slits that open already during buccal expansion, based on personal observations from video recordings (Fig. 1; Movie 1). This maintains suction force throughout the whole expansion sequence and even after maximum expansion is reached. This was notably measured in the tiger salamander (*A. tigrinum*) by Lauder and Shaffer (1986) who found that larval individuals generated significantly greater negative pressures than metamorphosed individuals. Thus, whereas metamorphs may rely on faster buccal expansion movements to increase their suction force, larvae may compensate for their slower movements and their increasing maximum gape through their labial lobes and gill slits.

### Prevention of prey escape may rely on different morphological features in larvae and metamorphs

Once the prey is inside the buccal cavity, mouth closure prevents prey escape, and elevation of the hyoid allows the water engulfed during capture to be expelled (Figs 3B and 4B; Deban, 2003; Toussaint-Lardé et al., 2025). These steps are challenging in metamorphs because the risk of prey escape increases. Indeed, in the absence of gill slits, once maximal buccal expansion is reached, water flow is reversed, which can facilitate prey escape (Movie 2). Moreover, the larger prey consumed by metamorphs are maintained by the jaws while the hyoid is being elevated to its resting position to expel water through the mouth (bidirectional flow system; Lauder and Shaffer, 1986; Deban, 2003). This can further increase the risk of prey escape (Movie 2). Yet, the slope for jaw closing duration (JCD) is 3.64 times greater in metamorphs than in larvae (Fig. 3B), and the slope for hyoid elevation duration (DhdE) is 21.98 times greater in metamorphs than in larvae (Fig. 4B). Thus, the faster rates of jaw closing and hyoid elevation in metamorphs compared to larvae may be advantageous by reducing the risk of prey escape through shorter durations of these steps.

In larvae, prey escape risks are likely reduced thanks to the labial lobes, which occlude the entire lateral margins of the mouth (Fig. 1; Fig. S1, Movie 1), and the gill slits, which maintain a suction flow towards the back of the head and draw prey deeper into the throat (Toussaint-Lardé et al., 2025). The beneficial role of gill slits was highlighted by Reilly and Lauder (1988) and Lauder and Reilly (1988), who reported a decrease in prey capture success when gill slits were resorbed in metamorphosed eastern newt (*N. viridescens*) and when gill slits were surgically sutured in the axolotl (*Ambystoma mexicanum*), respectively. Here, again, while metamorphs may rely on faster mouth closing and hyoid elevation, larvae may rely on their labial lobes and gill slits to reduce the risk of prey escape.

### Reduced interstage competition remains to be confirmed

From a functional perspective, except for the maximum gape of the mouth, our results indicate a decoupling between larval and metamorphic suction feeding, which is in line with the ontogenetic decoupling hypothesis. However, we cannot determine whether this decoupling effectively reduces interstage competition. However, the Gálvez et al. (2021) study may support the hypothesis of a reduction of interstage competition between larval and metamorphic Iberian ribbed newt. They investigated the effects of adult and larval *P. waltl* abundance on the population of other amphibians across multiple ponds. Their results revealed significant negative effects of *P. waltl* abundance on other amphibians present, while on the contrary, the presence of larval *P. waltl* was positively related to adult *P. waltl* abundance. However, further analyses are required to confirm this positive relationship between larvae and metamorphs of *P. waltl* and to determine whether the morpho-functional trade-off inferred here is real and, in this case, if it contributes to reduced interstage competition. For example, measuring intrabuccal pressure during suction and/or assessing prey capture success across different prey types (e.g. static versus evasive prey and prey taxa) could reveal whether larvae and metamorphs exhibit comparable performance and/or whether each life stage is specialized for particular prey categories. For now, Iberian ribbed newts are generally described as polyphagous opportunistic predators whose diet largely reflects prey availability (Garcia-de-Lomas et al., 2012; Salvador, 2014; Escoriza et al., 2020; Gálvez et al., 2021). However, Garcia-de-Lomas et al. (2012) reported that small larvae may preferentially consume calanoid copepods and ostracods over cyclopoids. Further field observations could clarify whether larvae and metamorphs display distinct prey preferences.

### Comparison with other salamander species

Surprisingly, Shaffer and Lauder (1988) found no effect of metamorphosis on aquatic feeding kinematics in larval and aquatic metamorphosed *A. tigrinum*. However, their methodology differed from ours, as individuals in the Shaffer and Lauder (1988) study were tested 3 months after metamorphosis to minimize size differences between life stages, whereas our analyses explicitly account for the effect of size and compared animals that strongly differed in size. Because individuals in their study were of similar size, their muscle mass was likely comparable, potentially explaining the absence of kinematic differences. It is therefore possible that we would have obtained similar results if we had compared larvae and metamorphs of similar size, as in Shaffer and Lauder's (1988) study.

In contrast, the results presented by Toussaint-Lardé et al. (2025) echo ours. Indeed, they reported faster buccal expansion, jaw closing, and hyoid elevation movements in adult *A. mexicanum* compared to larvae, which they also linked to the higher degree of cranial ossification in adults. However, these differences were detected in speed and acceleration of the movements and not in the durations. For instance, in their study, the TMG was constant irrespective of life stage and the duration of the gape (DG), and TMhd increased proportionally to size across all stages. In the Iberian ribbed newts, larvae and metamorphs show a reversed duration pattern with size (Figs 3 and 4). The fact that the differences in durations are more pronounced in the Iberian ribbed newt than in the axolotl may be due to differences in their life cycle: Iberian ribbed newts are biphasic and undergo complete metamorphosis, whereas axolotls are paedomorphic and undergo only partial metamorphosis. Therefore, head remodelling is more pronounced in the Iberian ribbed newt. Nevertheless, it is interesting to note that even paedomorphic species may not share the same feeding kinematics.

It should be noted, however, that both our study and that of Toussaint-Lardé et al. (2025) are subject to a lack of size overlap among life stages: adult and immature *A. mexicanum* did not overlap in size, nor did larval and metamorphic *P. waltl*. We thus cannot be certain that the estimated linear relationships between the kinematic variables and size would have remained the same if there had been greater size overlap between these stages. Nevertheless, in nature, adults and metamorphosed individuals are generally larger than immature and larval individuals, limiting the extent of size overlap between life stages.

### Conclusions and perspectives

To conclude, as expected and in line with the ontogenetic decoupling hypothesis, the majority of our kinematic variables scale differently with body size across developmental stage. More specifically, metamorphosis modifies the relationships between body size [snout–vent length (SVL)] and the duration of jaw and hyoid movements (TMG, JCD, TMhd, PCD, DG, DhdE; Figs 3 and 4), as well as the MHA (Fig. 2B) and MGA (Fig. 5) during suction feeding. From a functional perspective, this indicates a decoupling between larval and metamorphic suction feeding. However, we cannot determine whether this decoupling effectively reduces interstage competition as predicted by the ontogenetic decoupling hypothesis. Our results suggest rather a morpho-functional trade-off between larvae and metamorphs. The slower movements of larvae compared to metamorphs, when scaled to body size (with absolute slope estimates of durations between 1.67 and 21.98 times greater in metamorphs than in larvae; Figs 3 and 4), may be compensated for by the presence of labial lobes and gill slits, which likely enhance suction efficiency and reduce the risk of prey escape. In metamorphs, gill slits are lost, and labial lobes are reduced, but suction efficiency and prey capture success may instead benefit from cranial remodelling and increased ossification, enabling faster movements. To assess this morpho-functional trade-off, future studies should combine kinematic analyses with direct measurements of prey capture success, intraoral pressure during suction feeding events, and cranial ossification, all quantified across the same ontogenetic series.

## MATERIALS AND METHODS

### Specimens used

Animal handling and experiments followed Directive 2010/63/EU on the protection of animals used for scientific purposes within the European Union and were approved by the Comité d’Éthique Cuvier (project 2024-68-134). All animals came from breeding colonies in French laboratories (UMR 7179, CNRS–MNHN, Paris, France). All animals were housed under entirely aquatic conditions. Larvae are easily distinguishable from metamorphs because their morphology differs. Notably, larvae possess external gills, gill slits, extensive labial lobes that occlude the entire lateral portion of the mouth, and a tail fin. During metamorphosis, the external gills, gill slits, and tail fin are resorbed, and the labial lobes are markedly reduced in metamorphs (Fig. S1, Movies 1 and 2). The study included nine larvae and ten aquatic metamorphs of the Iberian ribbed newt (*P. waltl* Michahelles, 1830). However, four individuals were filmed at both developmental stages, resulting in a total of 15 biological individuals. Accordingly, each video sequence was assigned both a stage-specific identifier (L1–L9 for larvae and M1–M10 for metamorphs; Table 2; Table S1) and a biological identifier (ind1–ind15; Table 2; Table S1). Individuals ind5–ind8 were recorded at both stages, corresponding to larvae L5–L8 and metamorphs M3, M5, M1, and M2, respectively (Table 2; Table S1). SVL was used as a proxy for body size (Fig. S2A): larval SVL ranged from 1.80 to 3.50 cm, and metamorphic SVL ranged from 5.80 to 8.69 cm. The size and developmental stage of each individual are available in Table 2.

**Table 2.** Developmental stage, size, and number of sequences per individual

Developmental stage	Stage-specific ID	Biological ID	Snout–vent length (cm)	Number of sequences
Larvae	L1	ind1	1.80	11
	L2	ind2	1.80	10
	L3	ind3	1.80	10
	L4	ind4	2.00	10
	L5	ind5	2.10	8
	L6	ind6	2.20	11
	L7	ind7	2.20	12
	L8	ind8	2.60	12
	L9	ind9	3.50	12
Metamorphs	M1	ind7	5.80	4
	M2	ind8	5.90	5
	M3	ind5	6.50	6
	M4	ind10	6.86	5
	M5	ind6	6.90	5
	M6	ind11	7.02	5
	M7	ind12	7.08	3
	M8	ind13	7.43	5
	M9	ind14	7.90	2
	M10	ind15	8.69	7

ind5, ind6, ind7 and ind8 were filmed at both the larval and metamorphic stages.

### Experimental setup

Animals were placed in an experimental tank filled with water at ambient temperature (18°C). A checkerboard was added as a scale. To trigger feeding sequences, larvae L1–L9 and metamorphs M1–M3 and M5 were fed dead bloodworms, whereas metamorphs M4 and M6–M10 were given pieces of earthworm, as this was the diet to which they were accustomed in the breeding colonies. Two infrared lights (Dedolight LEDs) were used to record feeding sequences under low-light conditions without disturbing the animals. Feeding sequences were captured using a Phantom Miro R311 high-speed camera (1000 fps; 1200×800 pixels) with a Nikon 50 mm lens, positioned at the front of the tank and connected to a computer running PCC software (version 3.6). All feeding sequences were saved in ‘.cine’ format. Only the successful feeding sequences in which the animals were positioned in lateral view were retained for this study. A total of 96 feeding sequences from nine larvae and 47 from ten metamorphs were analysed (Table 2).

### Kinematic data extracted

We recorded a total of ten feeding kinematic variables. Eight of them were directly extracted from the video sequences by a single operator:

- MGA (°): defined as the angle between the upper jaw, the jaw joint, and the lower jaw at maximal mouth opening (Fig. S2B);
- TMG (ms): defined as the duration from the onset of mouth opening to maximal mouth opening;
- MG (cm): defined as the distance between the tips of the upper and lower jaws at maximal mouth opening (Fig. S2B);
- JCD (ms): defined as the duration from the maximal mouth opening to the closing of the jaws;
- MHA (°): defined as the difference between the initial and maximum angles formed by the upper jaw, the eye, and the shoulder during the feeding sequence (Fig. S2B);
- Mhd (cm): defined as the distance between the jaw joint and the posterior tip of the gular fold at maximal hyoid depression (Fig. S2B);
- TMhd (ms): defined as the duration from the onset of mouth opening to maximal hyoid depression;
- PCD (ms): defined as the duration from the onset of mouth opening to the end of hyoid elevation.

Two other variables were calculated using previously extracted variables:

- DG: defined as the duration from the onset of mouth opening to jaw closure, calculated as TMG+JCD;
- DhdE: defined as the time from Mhd to the time when the hyoid is back to its resting position, calculated as PCD−TMhd.

Values of the extracted kinematic variables are available in Table S1.

### Statistical design

All the statistical analyses were performed using R (v4.4.2; R Core Team, 2024, https://www.R-project.org//). The R script is available on GitHub (see ‘Data and resource availability’).

#### Statistical modelling of kinematic variables

All kinematic variables except MHA were log-transformed using the decadic logarithm (‘log10’ function; base R v4.4.2; R Core Team, 2024, https://www.R-project.org//). MHA was transformed using the natural logarithm of (1+*x*) (‘log1p’ function; base R v4.4.2; R Core Team, 2024, https://www.R-project.org//) to accommodate a zero value in the feeding sequence ‘indiv n5 vid 8’ (row 103 of Table S1) from larva L5. SVL was transformed using the same logarithmic transformation as the corresponding response variable (i.e. ‘log10’ for all variables except MHA and ‘log1p’ for MHA).

Each kinematic variable was analysed using a linear mixed-effects model including two random effects. The first random effect, *(1 | stage-specific identifier)*, accounted for the repeated feeding sequences recorded from the same individual within each developmental stage (i.e. pseudoreplication). The second random effect, *(1 | biological identifier)*, accounted for the fact that individuals ind5–ind8 were recorded at both the larval and metamorphic stages. The fixed effects of the model included (1) body size (SVL), (2) developmental stage, and (3) their interaction, as size and developmental stage may have a joint effect on the kinematic variables. Models were fitted by maximum likelihood using the ‘lmer’ function (lmerTest v3.1.3; Kuznetsova et al., 2017). The corresponding R formula syntax was as follows: *Kinematic variable∼developmental stage×SVL+(1 | stage-specific identifier)+(1 | biological identifier)*. Model residual normality was assessed visually using Q-Q plots generated with the ‘ggqqplot’ function (ggpubr v0.6.2, Kassambara, 2025, https://CRAN.R-project.org/package=ggpubr; Fig. S3). Summaries of the mixed-effects model estimates were obtained using the ‘tab_model’ function (sjPlot v2.9.0, Lüdecke, 2025, https://CRAN.R-project.org/package=sjPlot; Table S2).

#### Statistical testing

Type II ANOVAs with Satterthwaite's method were performed on each linear mixed-effects model in order to test whether size, developmental stage, and their interaction (explanatory variables) affected the kinematic variables (response variables), using the ‘anova’ function (lmerTest v3.1.3; Kuznetsova et al., 2017). For the variables MG and Mhd, as the interaction term between SVL and developmental stage was not significant, reduced linear mixed-effects models excluding this interaction term were fitted using the following formula: *Kinematic variable∼developmental stage+SVL+(1 | stage-specific identifier)+(1 | biological identifier).* These reduced models provided a better fit to the data according to AIC and BIC (Table 1). AIC and BIC values are provided by the ‘summary’ function (R Core Team, 2024, https://www.R-project.org//). New type II ANOVAs were subsequently performed on these reduced models to assess the independent effects of body size and developmental stage on MG and Mhd.

#### Linear regressions

To visualize the results of the ANOVAs and the estimates of the model, model-based predictions of kinematic variables across body size were generated using the ‘ggpredict’ function (ggeffects v2.1.0; Lüdecke, 2018). These predictions are derived from the fixed-effects estimates of the linear mixed-effects models, which account for repeated measurements within individuals. Predicted values were then visualized using the ggplot2 package (v3.5.2; Wickham, 2016). When only SVL had a significant effect on kinematic variables, predictions were plotted across the full dataset. When developmental stage, or its interaction with body size, was significant, predictions were plotted separately for larval and metamorphic stages. Slope and intercept estimate details were extracted from the mixed-effects models using the functions ‘emtrends’ and ‘emmeans’ (emmeans v2.0.1; Russell et al., 2025, https://CRAN.R-project.org/package=emmeans). Kinematic variables were considered to exhibit a significant negative relationship with size if the upper bound of the 95% confidence interval (CI) was below zero. Conversely, they were considered to exhibit a significant positive relationship if the lower bound of the 95% CI was above zero. If the 95% CI included zero, the relationship was not significant. Raw data were superimposed on the model-predicted regression lines to allow visual assessment of whether the fitted relationships are consistent with the observed data.

### AI use

ChatGPT was used to help write part of the R script and to correct the English wording of some sentences. The authors subsequently reviewed and edited the content as necessary and take full responsibility for the publication’s final content.

## Supplementary Material



10.1242/biolopen.062752_sup1 Supplementary information

Table S1. Dataset used in the analyses.

Table S2. Summary of the linear mixed-effects models for the different kinematic variables. For MG and Mhd, both a full model (including the interaction between size and developmental stage) and a reduced model (excluding the interaction term) are presented, as the interaction term did not significantly affect these variables. Raw data were transformed using the decadic logarithm ('log10' transformation), except for the MHA analysis, for which the natural logarithm of 1 + x ('log1p' transformation) was used.

## References

[BIO062752C1] Ashley-Ross, M. A. (1994). Metamorphic and speed effects on hindlimb kinematics during terrestrial locomotion in the salamander *Dicamptodon tenebrosus*. *J. Exp. Biol.* 193, 285-305. 10.1242/jeb.193.1.285 9317817

[BIO062752C2] Azizi, E. and Landberg, T. (2002). Effects of metamorphosis on the aquatic escape response of the two-lined salamander (*Eurycea bislineata*). *J. Exp. Biol.* 205, 841-849. 10.1242/jeb.205.6.841 11914392

[BIO062752C3] Belles, X. (2023). Investigating the origin of insect metamorphosis. *eLife* 12, e94410. 10.7554/eLife.94410 PMC10735215 38126357

[BIO062752C4] Brown, J. H., West, G. B. and Enquist, B. J. (2000). Scaling in biology: patterns and processes, causes and consequences. In *Scaling in Biology* (ed. J. H. Brown and G. B. West), pp. 1-24. New York: Oxford University Press.

[BIO062752C5] Carrier, D. R. (1996). Ontogenetic limits on locomotor performance. *Physiol. Zool.* 69, 467-488. 10.1086/physzool.69.3.30164211

[BIO062752C6] China, V. and Holzman, R. (2014). Hydrodynamic starvation in first-feeding larval fishes. *Proc. Natl. Acad. Sci. USA* 111, 8083-8088. 10.1073/pnas.1323205111 PMC4050599 24843180

[BIO062752C7] Currey, J. D. (2002). *Bones: Structure and Mechanics*. Princeton, NJ: Princeton University Press. http://www.jstor.org/stable/j.ctt4cg9wv.

[BIO062752C8] Deban, S. M. (2003). Constraint and convergence in the evolution of salamander feeding. In *Vertebrate Biomechanics and Evolution* (ed. V. L. Bels, J. P. Gasc and A. Casinos), pp. 161-178. Oxford, UK: BIOS Scientific Publishers.

[BIO062752C9] Deban, S. M. and Marks, S. B. (2002). Metamorphosis and evolution of feeding behaviour in salamanders of the family Plethodontidae. *Zool. J. Linn. Soc.* 134, 375-400. 10.1046/j.1096-3642.2002.00004.x

[BIO062752C10] Ebenman, B. (1992). Evolution in organisms that change their niches during the life cycle. *Am. Nat.* 139, 990-1021. 10.1086/285370

[BIO062752C11] Escoriza, D., Ben Hassine, J., Boix, D. and Sala, J. (2020). Diet of larval *Pleurodeles waltl* (Urodela: Salamandridae) throughout its distributional range. *Limnetica.* 39, 667-676. 10.23818/limn.39.43

[BIO062752C12] Ferry-Graham, L. A. (1998). Effects of prey size and mobility on prey-capture kinematics in leopard sharks *Triakis semifasciata*. *J. Exp. Biol.* 201, 2433-2444. 10.1242/jeb.201.16.2433 9679105

[BIO062752C13] Gálvez, Á., Mesquita-Joanes, F. and Monrós, J. S. (2021). Are Iberian ribbed newts *Pleurodeles waltl* Michahelles, 1830 negatively affecting the populations of other amphibians? *Ecol. Res.* 36, 389-400. 10.1111/1440-1703.12203

[BIO062752C14] Garcia-de-Lomas, J., Torres, J. M., Reques, R. and Hernández, I. (2012). *Pleurodeles waltl* newt larvae diet regarding available prey: choice or mere coincidence? *Copeia* 2012, 756-761. 10.1643/CE-11-032

[BIO062752C15] García-Paris, M. (1985). *Los anfibios de España*. Madrid: Publicaciones de Extensión Agraria.

[BIO062752C16] Geffen, A. J., van der Veer, H. W. and Nash, R. D. M. (2007). The cost of metamorphosis in flatfishes. *J. Sea. Res.* 58, 35-45. 10.1016/j.seares.2007.02.004

[BIO062752C17] Heiss, E. and Grell, J. (2019). Same but different: aquatic prey capture in paedomorphic and metamorphic alpine newts. *Zool. Lett.* 5, 24. 10.1186/s40851-019-0140-4 PMC6660708 31372238

[BIO062752C18] Heiss, E., Natchev, N., Gumpenberger, M., Weissenbacher, A. and Van Wassenbergh, S. (2013). Biomechanics and hydrodynamics of prey capture in the Chinese giant salamander reveal a high-performance jaw-powered suction feeding mechanism. *J. R. Soc. Int.* 10, 20121028. 10.1098/rsif.2012.1028 PMC3627076 23466557

[BIO062752C19] Heiss, E., Aerts, P. and Van Wassenbergh, S. (2015). Flexibility is everything: prey capture throughout the seasonal habitat switches in the smooth newt *Lissotriton vulgaris*. *Org. Divers. Evol.* 15, 127-142. 10.1007/s13127-014-0187-1 PMC4470538 26097413

[BIO062752C64] Hernández, L. P. (2000). Intraspecific scaling of feeding mechanics in an ontogenetic series of zebrafish, *Danio rerio*. *J. Exp. Biol.* 203, 3033-3043. 10.1242/jeb.203.19.3033 10976040

[BIO062752C20] Herrel, A. and Gibb, A. C. (2006). Ontogeny of performance in vertebrates. *Physiol. Biochem. Zool.* 79, 1-6. 10.1086/498196 16380923

[BIO062752C21] Istock, C. A. (1967). The evolution of complex life cycle phenomena: an ecological perspective. *Evolution* 21, 592-605. 10.1111/j.1558-5646.1967.tb03414.x 28563694

[BIO062752C22] Johansson, F., Lederer, B. and Lind, M. I. (2010). Trait performance correlations across life stages under environmental stress conditions in the common frog, *Rana temporaria*. *PLoS ONE* 5, e11680. 10.1371/journal.pone.0011680 PMC2908130 20657779

[BIO062752C23] Joven, A., Kirkham, M. and Simon, A. (2015). Husbandry of Spanish ribbed newts (*Pleurodeles waltl*). In *Salamanders in Regeneration Research. Methods in Molecular Biology* (ed. A. Kumar and A. Simon), pp. 47-70. New York, NY: Springer. 10.1007/978-1-4939-2495-0_4 25740476

[BIO062752C24] Keast, A. (1985). The piscivore feeding guild of fishes in small freshwater ecosystems. *Environ. Biol. Fish.* 12, 119-129. 10.1007/BF00002764

[BIO062752C25] Koumoundouros, G., Ashton, C., Xenikoudakis, G., Giopanou, I., Georgakopoulou, E. and Stickland, N. (2009). Ontogenetic differentiation of swimming performance in gilthead seabream (*Sparus aurata*, Linnaeus 1758) during metamorphosis. *J. Exp. Mar. Biol. Ecol.* 370, 75-81. 10.1016/j.jembe.2008.12.001

[BIO062752C26] Kusano, T., Kusano, H. and Miyashita, K. (1985). Size-related cannibalism among larval *Hynobius nebulosus*. *Copeia* 1985, 472-476. 10.2307/1444861

[BIO062752C27] Kuznetsova, A., Brockhoff, P. B. and Christensen, R. H. B. (2017). lmerTest package: tests in linear mixed effects models. *J. Stat. Soft.* 82, 1-26. 10.18637/jss.v082.i13

[BIO062752C28] Lauder, G. V. and Reilly, S. M. (1988). Functional design of the feeding mechanism in salamanders: causal bases of ontogenetic changes in function. *J. Exp. Biol.* 134, 219-233. 10.1242/jeb.134.1.219

[BIO062752C29] Lauder, G. V. and Shaffer, H. B. (1986). Functional design of the feeding mechanism in lower vertebrates: unidirectional and bidirectional flow systems in the tiger salamander. *Zool. J. Linn. Soc.* 88, 277-290. 10.1111/j.1096-3642.1986.tb01191.x

[BIO062752C30] Laudet, V. (2011). The origins and evolution of vertebrate metamorphosis. *Curr. Biol.* 21, R726-R737. 10.1016/j.cub.2011.07.030 21959163

[BIO062752C31] Lüdecke, D. (2018). ggeffects: tidy data frames of marginal effects from regression models. *JOSS* 3, 772. 10.21105/joss.00772

[BIO062752C32] Lukanov, S., Tzankov, N., Handschuh, S., Heiss, E., Naumov, B. and Natchev, N. (2016). On the amphibious food uptake and prey manipulation behavior in the Balkan-Anatolian crested newt (*Triturus ivanbureschi*, Arntzen and Wielstra, 2013). *Zoology* 119, 224-231. 10.1016/j.zool.2016.02.002 27013264

[BIO062752C33] Manthey, C., Metcalf, C. J. E., Monaghan, M. T., Steiner, U. K. and Rolff, J. (2024). Rapid growth and the evolution of complete metamorphosis in insects. *Proc. Natl. Acad. Sci. USA* 121, e2402980121. 10.1073/pnas.2402980121 PMC11420152 39250668

[BIO062752C34] Moran, N. A. (1994). Adaptation and constraint in the complex life cycles of animals. *Annu. Rev. Ecol. Syst.* 25, 573-600. 10.1146/annurev.es.25.110194.003041

[BIO062752C35] O'Reilly, J. C., Lindstedt, S. L. and Nishikawa, K. C. (1993). The scaling of feeding kinematics in toads (Anura: Bufonidae). *Am. Zool.* 33, 147A.

[BIO062752C36] Padilla, P., Herrel, A. and Denoël, M. (2024). Invading new climates at what cost? Ontogenetic differences in the thermal dependence of metabolic rate in an invasive amphibian. *J. Therm. Biol.* 121, 103836. 10.1016/j.jtherbio.2024.103836 38604116

[BIO062752C37] Raffaëlli, J. (2007). *Les Urodèles du monde*. Plumelec, France: Penclen edition.

[BIO062752C38] Reilly, S. M. (1996). The metamorphosis of feeding kinematics in *Salamandra salamandra* and the evolution of terrestrial feeding behavior. *J. Exp. Biol.* 199, 1219-1227. 10.1242/jeb.199.5.1219 9319073

[BIO062752C39] Reilly, S. M. and Lauder, G. V. (1988). Ontogeny of aquatic feeding performance in the eastern newt, *Notophthalmus viridescens* (Salamandridae). *Copeia.* 1988, 87-91. 10.2307/1445926

[BIO062752C40] Richard, B. A. and Wainwright, P. C. (1995). Scaling the feeding mechanism of largemouth bass (*Micropterus salmoides*): kinematics of prey capture. *J. Exp. Biol.* 198, 419-433. 10.1242/jeb.198.2.419

[BIO062752C41] Robinson, M. P. and Motta, P. J. (2002). Patterns of growth and the effects of scale on the feeding kinematics of the nurse shark (*Ginglymostoma cirratum*). *J. Zool.* 256, 449-462. 10.1017/S0952836902000493

[BIO062752C42] Salvador, A. (2014). Gallipato – *Pleurodeles waltl* Michahelles, 1830. In *Enciclopedia Virtual de los Vertebrados Españoles* (ed. A. Salvador and I. Martínez-Solano). Madrid, Spain: Museo Nacional de Ciencias Naturales. http://www.vertebradosibericos.org/.

[BIO062752C43] Schmidt-Nielsen, K. (1984). *Scaling: Why* *I**s Animal Size* *S**o Important?* Cambridge, UK: Cambridge University Press.

[BIO062752C44] Shaffer, H. B. and Lauder, G. V. (1988). The ontogeny of functional design: metamorphosis of feeding behaviour in the tiger salamander (*Ambystoma tigrinum*). *J. Zool.* 216, 437-454. 10.1111/j.1469-7998.1988.tb02440.x

[BIO062752C45] Shaffer, H. B., Austin, C. C. and Huey, R. B. (1991). The consequences of metamorphosis on salamander (*Ambystoma*) locomotor performance. *Physiol. Zool.* 64, 212-231. 10.1086/physzool.64.1.30158520

[BIO062752C46] Smirnov, S. V., Merkulova, K. M. and Vassilieva, A. B. (2020). Skull development in the Iberian newt, *Pleurodeles waltl* (Salamandridae: Caudata: Amphibia): timing, sequence, variations, and thyroid hormone mediation of bone appearance. *J. Anat.* 237, 543-555. 10.1111/joa.13210 PMC7476196 32412118

[BIO062752C47] Sparreboom, M. (2014). *Salamanders of the Old World*. Zeist, Netherlands: KNNV Publishing.

[BIO062752C48] Ten Brink, H. (2018). Evolution of complex life cycles. *PhD thesis*, University of Amsterdam, Amsterdam, Netherlands. https://dare.uva.nl/id/dc234b3e-1ea6-426c-8087-440302433324.

[BIO062752C49] Ten Brink, H., De Roos, A. M. and Dieckmann, U. (2019). The evolutionary ecology of metamorphosis. *Am. Nat.* 193, E116-E131. 10.1086/701779 31002571

[BIO062752C50] Thompson, B. J. (2021). From genes to shape during metamorphosis: a history. *Curr. Opin. Insect. Sci.* 43, 1-10. 10.1016/j.cois.2020.08.008 32898719

[BIO062752C51] Toussaint-Lardé, I., Louppe, V., Sanchez-Garrido, F., Taillades, M., Amine, R., Mandica, M., Heude, E., Herrel, A. and Fabre, A.-C. (2025). The impact of size and ontogeny on suction feeding kinematics in the axolotl (*Ambystoma mexicanum*). *Biol. Open.* 14, bio061860. 10.1242/bio.061860 PMC12309904 40679315

[BIO062752C52] Van Wassenbergh, S. and Heiss, E. (2016). Phenotypic flexibility of gape anatomy fine-tunes the aquatic prey-capture system of newts. *Sci. Rep.* 6, 29277. 10.1038/srep29277 PMC4935879 27383663

[BIO062752C53] Wainwright, P. C. and Richard, B. A. (1995). Predicting patterns of prey use from morphology of fishes. *Environ. Biol. Fish.* 44, 97-113. 10.1007/BF00005909

[BIO062752C54] Wainwright, P. K. and Shaw, S. S. (1999). Morphological basis of kinematic diversity in feeding sunfishes. *J. Exp. Biol.* 202, 3101-3110. 10.1242/jeb.202.22.3101 10539958

[BIO062752C55] Wainwright, P., Carroll, A. M., Collar, D. C., Day, S. W., Higham, T. E. and Holzman, R. A. (2007). Suction feeding mechanics, performance, and diversity in fishes. *Integr. Comp. Biol.* 47, 96-106. 10.1093/icb/icm032 21672823

[BIO062752C65] Wainwright, P. C. and Day, S. W. (2007). The forces exerted by aquatic suction feeders on their prey. *J. R. Soc. Interface* 4, 553-560. 10.1098/rsif.2006.0197 PMC2373408 17251163

[BIO062752C56] Wassersug, R. J. and Sperry, D. G. (1977). The relationships of locomotion to differential predation on *Pseudacris triseriata* (Anura: Hylidae). *Ecology* 58, 830-839. 10.2307/1936218

[BIO062752C57] Watkins, T. B. (2001). A quantitative genetic test of adaptive decoupling across metamorphosis for locomotor and life-history traits in the Pacific tree frog, *Hyla regilla*. *Evolution* 55, 1668-1677. 10.1111/j.0014-3820.2001.tb00686.x 11580026

[BIO062752C58] Werner, E. E. (1974). The fish size, prey size, handling time relation in several sunfishes and some implications. *J. Fish. Res. Bd. Can.* 31, 1531-1536. 10.1139/f74-186

[BIO062752C59] Werner, E. E. (1988). Size, scaling, and the evolution of complex life cycles. In *Size-Structured Populations: Ecology and Evolution* (ed. E. Ebenman and L. Persson), pp. 60-81. Berlin, Germany: Springer.

[BIO062752C60] Whiteman, H. H., Wissinger, S. A. and Brown, W. S. (1996). Growth and foraging consequences of facultative paedomorphosis in the tiger salamander, *Ambystoma tigrinum nebulosum*. *Evol. Ecol.* 10, 433-446. 10.1007/BF01237728

[BIO062752C61] Wickham, H. (2016). *g**gplot2:* *Elegant Graphics for Data Analysis*. Cham, Switzerland: Springer International Publishing. https://scispace.com/pdf/ggplot2-elegant-graphics-for-data-analysis-102a7agfxj.pdf.

[BIO062752C62] Williams, P. J. and Brown, J. A. (1992). Development changes in the escape response of larval winter flounder *Pleuronectes americanus* from hatch through metamorphosis. *Mar. Ecol. Prog. Ser.* 88, 185-193. 10.3354/meps088185

[BIO062752C63] Wilson, R. S. (2005). Consequences of metamorphosis for the locomotor performance and thermal physiology of the newt *Triturus cristatus*. *Physiol. Biochem. Zool.* 78, 967-975. 10.1086/432923 16228936

