# Supplementary material for: The impact of metamorphosis and size on the aquatic feeding kinematics of the Iberian ribbed newt (Pleurodeles waltl Michahelles, 1830)
Source: Biol Open. 2026 Sep 28;15(9):bio062752. doi: 10.1242/bio.062752 (PMC13670357; doi:10.1242/bio.062752)
Supplement: Supplementary file 1 [file biolopen-15-062752-s1.pdf]

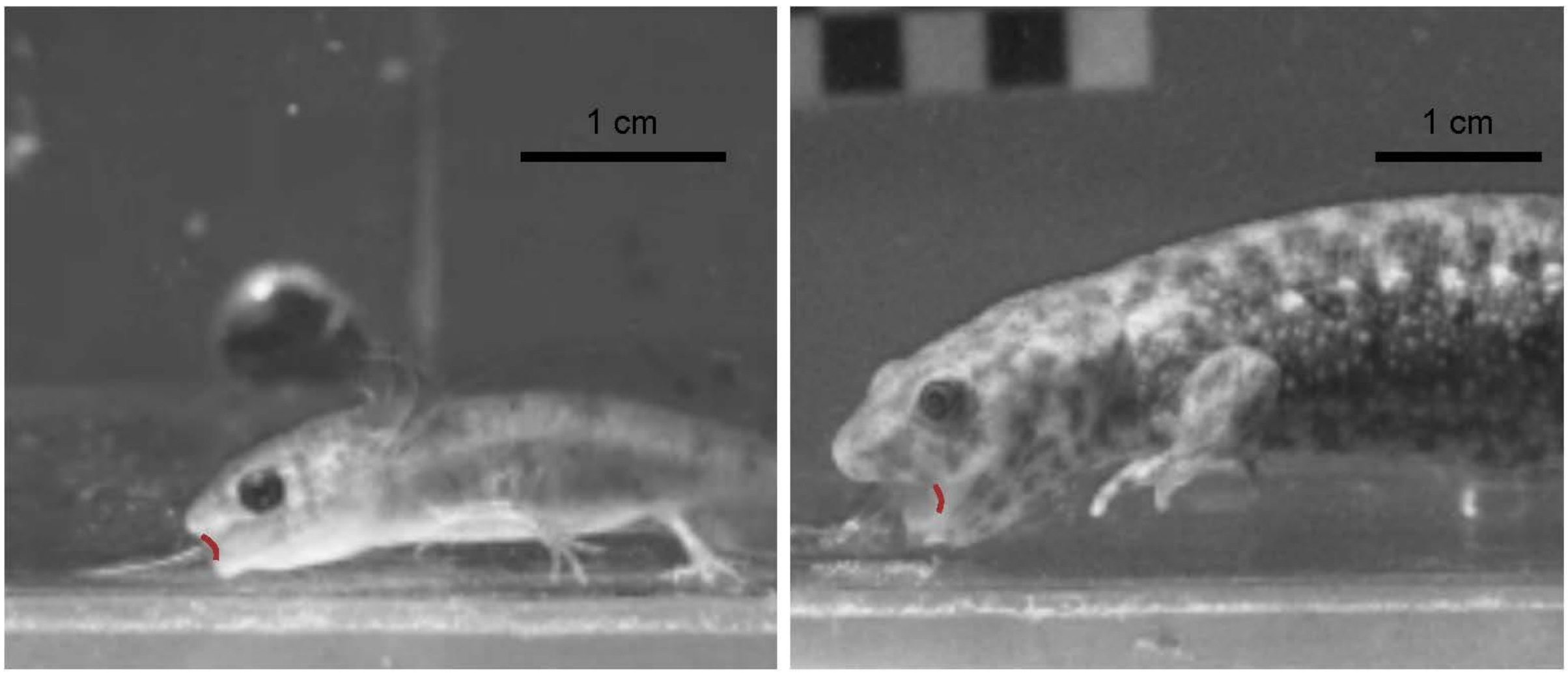

Fig. S1. Morphological differences in labial lobes across developmental stages. Photographs of an Iberian ribbed newt larva (left) and metamorph (right) at maximum gape distance (MG). The anterior margin of the labial lobe is highlighted in red. Labial lobes are markedly reduced after metamorphosis, maximally reaching the level of the eyes. Photographs were extracted from Videos S1 and S2.

Biology Open • Supplementary information

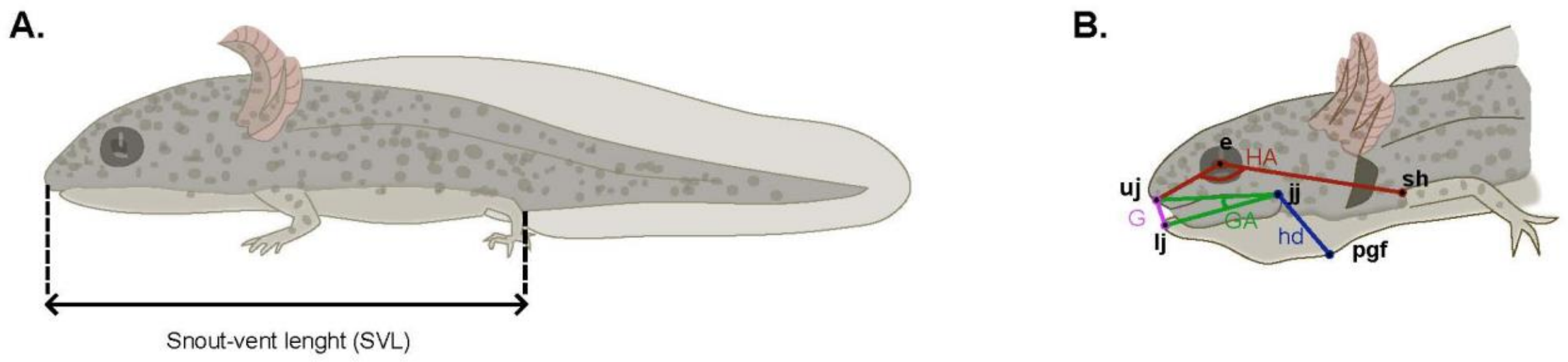

Fig. S2. Measurements performed on individuals. A. Snout–vent length (SVL, cm), measured as the distance from the tip of the snout to the posterior end of the cloacal slit (vent). B. Anatomical landmarks used to measure distances and angles. Uj, tip of the upper jaw; lj, tip of the lower jaw; jj, jaw joint; e, eye; sh, shoulder; pgf, posterior tip of the gular fold. G (cm), gape, defined as the distance between the tips of the upper and lower jaws; hd (cm), hyoid depression, measured as the distance between the jaw joint and the posterior tip of the gular fold; HA (°), head angle, defined as the angle formed by the upper jaw, eye, and shoulder; and GA (°), gape angle, defined as the angle between the upper jaw, jaw joint, and lower jaw. For illustrative purposes, G, hd, HA and GA are shown simultaneously. However, our analyses are based on the maximum value of each of these variable (maximum gape distance, MG; maximum hyoid depression, Mhd; maximum gape angle, MGA; and maximum head angle, MHA). Except for MG and MGA, these maxima do not occur simultaneously.

Biology Open • Supplementary information

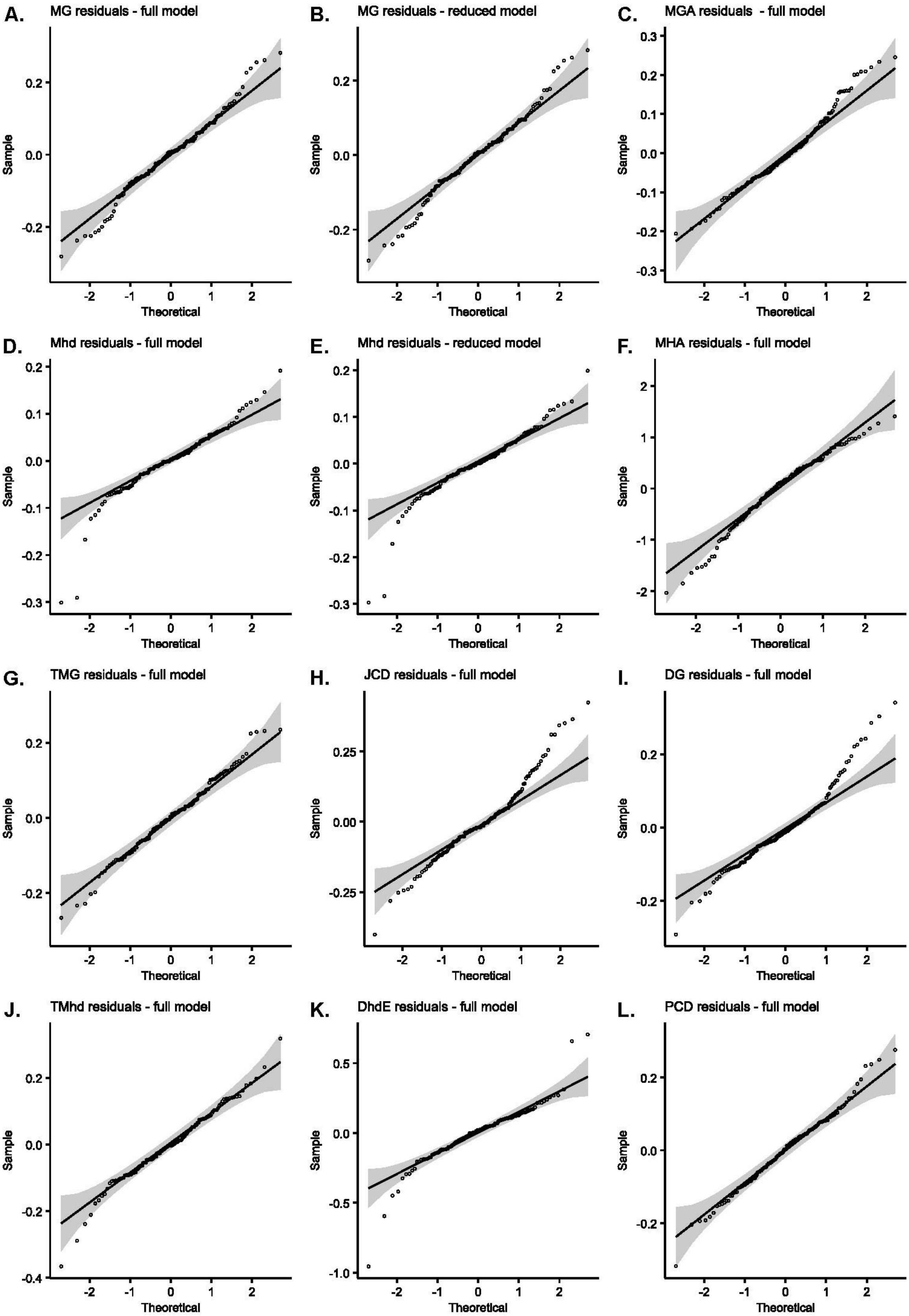

Biology Open • Supplementary information

Fig. S3. Normal Q-Q plots of residuals from the linear mixed-effects models. Data were log-transformed prior to model fitting to improve normality of residuals using the decadic logarithm (*log10*), except for the model of maximum head angle (MHA), for which data were transformed using the natural logarithm of *(1 + x)* (*log1p* transformation). Panels show residuals for: A–B, maximum gape distance (MG); C, maximum gape angle (MGA); D–E, maximum hyoid depression (Mhd); F, maximum head angle (MHA); G, time to maximum gape (TMG); H, jaw closing duration (JCD); I, gape duration (DG); J, time to maximum hyoid depression (TMhd); K, duration of hyoid elevation (DhdE); and L, prey capture duration (PCD). "Full model" indicates that the interaction between size and developmental stage was included, whereas "reduced model" indicates that this interaction was excluded. Only MG and Mhd were analysed using both full and reduced models; all other variables were analysed using the full model only. Globally, residuals are normally distributed; although, some models show excess kurtosis, with deviations at the tails from the theoretical normal quantiles. Such deviations may reduce statistical power and increase the risk of failing to detect significant effects.

Table S1. Dataset used in the analyses.

Available for download at
https://journals.biologists.com/bio/article-lookup/doi/10.1242/bio.062752#supplementary-data

Table S2. Summary of the linear mixed-effects models for the different kinematic variables. For MG and Mhd, both a full model (including the interaction between size and developmental stage) and a reduced model (excluding the interaction term) are presented, as the interaction term did not significantly affect these variables. Raw data were transformed using the decadic logarithm ('log10' transformation), except for the MHA analysis, for which the natural logarithm of 1 + x ('log1p' transformation) was used.

Available for download at
https://journals.biologists.com/bio/article-lookup/doi/10.1242/bio.062752#supplementary-data

Biology Open • Supplementary information

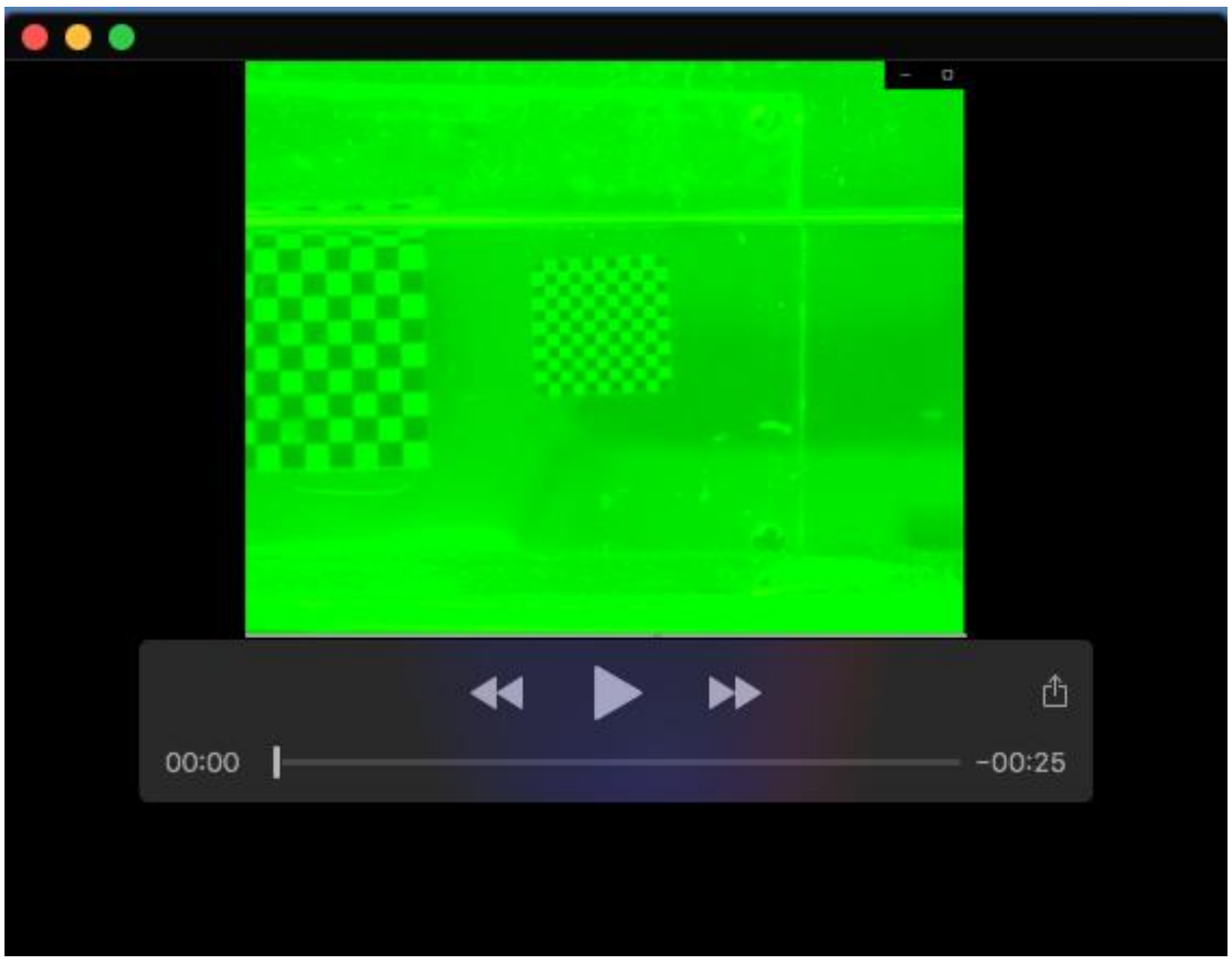

Movie 1. Typical suction feeding at larval stage. The squares on the largest checkerboard (left) are 0.5 cm wide. (field title: MS1_indiv_n6_vid6_larva_cropped.mp4; 1000 fps)

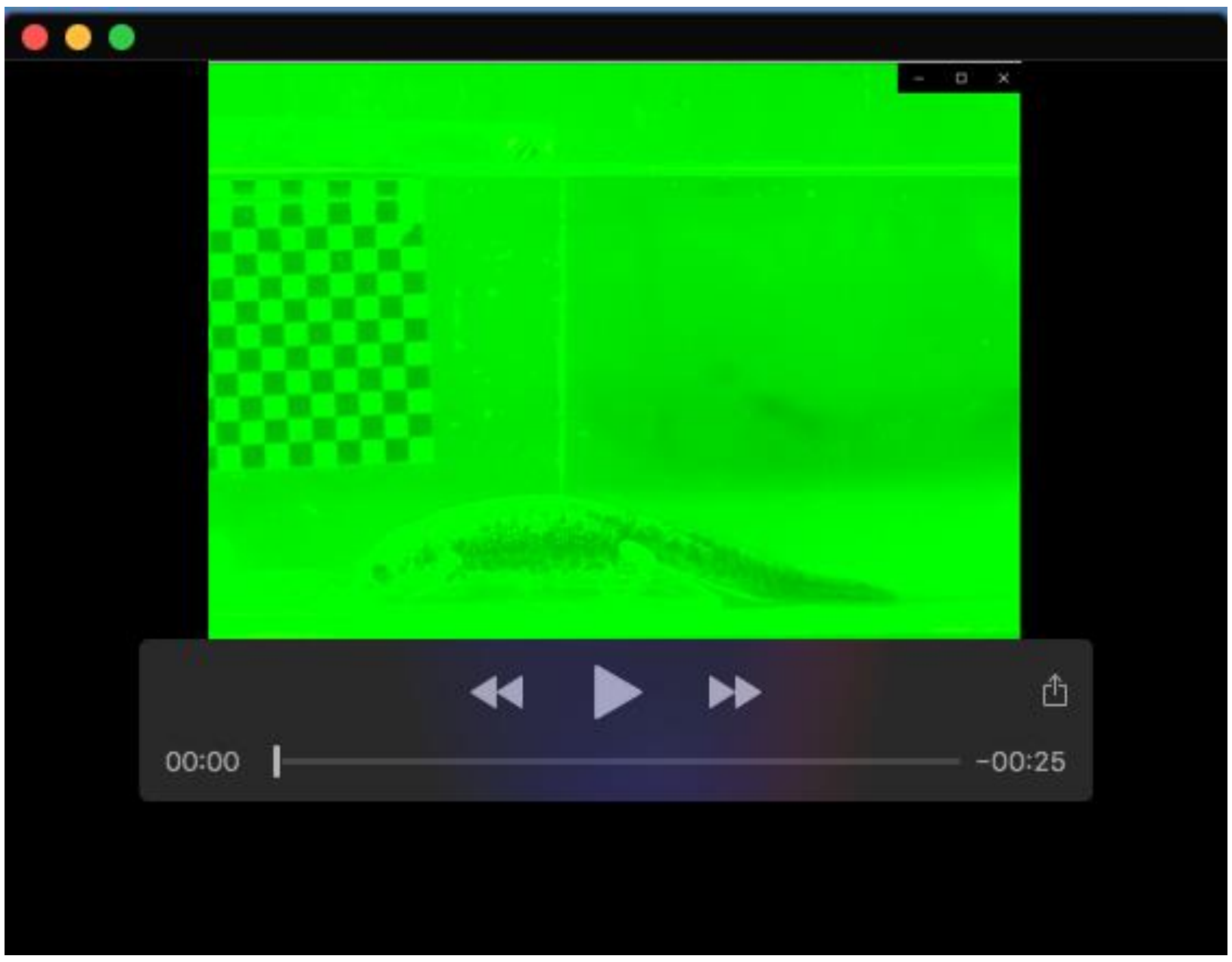

Movie 2. Typical suction feeding at metamorphic stage. The squares on checkerboard are 0.5 cm wide. (field title: MS2_indiv_n2_vid1_metamorph_cropped.mp4; 1000 fps)

Biology Open • Supplementary information
