# Supplementary material for: The impact of metamorphosis and size on the aquatic feeding kinematics of the Iberian ribbed newt (Pleurodeles waltl Michahelles, 1830)
Source: Biol Open. 2026 Sep 28;15(9):bio062752. doi: 10.1242/bio.062752 (PMC13670357; doi:10.1242/bio.062752)
Supplement: Supplementary file 2 [file biolopen-15-062752_review_history.pdf]

The impact of metamorphosis and size on the aquatic feeding kinematics of the Iberian ribbed newt (Pleurodeles waltl Michahelles, 1830)

Merlin Hugon-Daune-Fäs, Anthony Herrel, Anne-Claire Fabre and Isabelle Toussaint-Lardé
10.1242/bio.062752

Editor: Nicholas Wu

Review timeline

Original submission:	15 June 2026
Editorial decision:	19 June 2026
First revision received:	28 July 2026
Editorial decision:	2 August 2026
Second revision received:	26 August 2026
Accepted:	28 August 2026

Original submission

First decision letter

MS ID#: bio.062752

MS Title: The impact of metamorphosis and size on the aquatic feeding kinematics of the Iberian ribbed newt (Pleurodeles waltl Michahelles, 1830)

Authors: Isabelle Toussaint-Lardé; Merlin Hugon-Daune-Fäs; Anthony Herrel; Anne-Claire Fabre

I have now reached a decision on the above manuscript.

The reviewer reports are shown at the bottom of this email.

As you will see, the reviewers raised a number of substantial criticisms that prevent me from accepting the paper at this stage. Both reviewers acknowledged that the study is interesting. They have provided detailed reviews that mainly focus on clarity of the concepts (e.g. decoupling hypothesis, phenotypical plasticity, developmental trends), methodology (e.g., statistical justification and transparency), presentation of results, and paragraph structuring. Overall, they have not identified major conceptual or methodological flaws, thus if the authors are able to both reviewers' concerns, I would be willing to have the paper reviewed again.

At this stage, we also ask you to ensure your manuscript complies with our formatting guidelines. Provided you are able to fully address the referees' comments, we are positive about publication of your paper (we accept over 95% of revision submissions) and therefore hope you won't mind any extra work involved in reformatting your manuscript at this point.

Please upload both a 'clean' version of your Word file, along with a highlighted version clearly showing where you have made changes in the revised manuscript. Please avoid using 'Track changes' in Word files as these are lost in PDF conversion.

© 2026. Published by The Company of Biologists under the terms of the Creative Commons Attribution License (https://creativecommons.org/licenses/by/4.0/).

I should be grateful if you would also provide a point-by-point response detailing how you have dealt with the points raised by the reviewers in the 'Response to Reviewers' box. Please attend to all of the reviewers' comments. If you do not agree with any of their criticisms or suggestions please explain clearly why this is so.

Reviewer 1

Comments for the author

I thoroughly enjoyed reading this article. The paper describes the use of a series of high speed videos to characterise the kinematics of suction flow, specifically comparing larvae against metamorphs over a range of metrics. The work brings new information to the public domain and should be published after a revision that addresses the weaknesses. These are listed individually below:

The abstract is currently ca. 40 words over 200 words (see guidelines here: https://journals.biologists.com/bio/pages/manuscript-prep#section-3-2) - the authors would need to abridge some of the text.

I did not see a summary statement (see here: https://journals.biologists.com/bio/pages/manuscript-prep#section-3-2) - though this might only be needed if the paper is accepted.

The authors have introduced the paper quite well, but I think there are some minor improvements needed here to broaden the readership value by improving on certain details. The authors state for example that, "The main hypotheses supporting this, is the 'adaptive' (or ontogenetic) decoupling hypothesis according to which, metamorphosis breaks the correlations among the phenotypic traits related to fitness expressed at the different life stages (Istock, 1967; Moran, 1994, Ten Brink et al., 2019)." - I would like the authors to provide some detail preceding this sentence to introduce the decoupling hypothesis more effectively, since the text transitions directly from a statement on the benefits of metamorphosis outweighing its costs. Could the authors provide a better transition by discussing this aspect in more detail (needs only a few sentences in my opinion, but flow is key and this transition is too abrupt). The decoupling hypothesis is again mentioned in line 126, so it is important that the reader fully understands what this hypothesis entails.

I find the text immediately after the same sentence reproduced above is also a little too presumptive in how it provides detail and context, i.e. straight after "The main hypotheses supporting this, is the 'adaptive' (or ontogenetic) decoupling hypothesis according to which, metamorphosis breaks the correlations among the phenotypic traits related to fitness expressed at the different life stages (Istock, 1967; Moran, 1994, Ten Brink et al., 2019)." in lines 51-56. I would like to suggest that the authors rewrite this section so that it (a) reads more clearly and (b) provides a little further details on things like "each stage" and how exactly post-metamorphic life stages can develop independently from pre-metamorphic stages leading to reduced "inter-stage competition". This section feels like it needs better flow and explanation as it the way it currently reads, doesn't lead the reader in any particular direction as these terminologies are very generic. In some ways a lot of this is easier to understand in the proceeding paragraph.

The proceeding paragraph is very good but the "partial decoupling" mentioned on line 80 is treated again, somewhat too frivolously, so I would ask the authors to clarify what a partial decoupling entails here in better detail. These details in the introductory text are important, as the set the scene for the rest of the paper and the introduction "almost" does that but not quite effectively enough.

The last two paragraphs of the introduction need to be reordered more sensibly. The first of the two starts with aims (line 103) and then there is a paragraph (starting line 124) that is two lines long describing expectations. Please rearrange this such that there is only one paragraph, the research gap is properly identified (leading in from the details provided in preceding paragraphs and details available within these paragraphs), the hypothesis of the authors' paper is stipulated clearly (merge the expectations into this perhaps?), followed by the aims of the present paper. This

© 2026. Published by The Company of Biologists under the terms of the Creative Commons Attribution License (https://creativecommons.org/licenses/by/4.0/).

can then be followed by text stipulating how (generally) the authors will enables these aims and how they expect to be able thence, to accept or reject their hypothesis/expectations.

Biology Open as far as I know, usually places the results immediately after the introduction, followed by discussion and the methods are at the end. The results therefore need to be written in a way that flows from an introduction and the authors should format/revise the textual flow of the results section in order to accommodate this. These details are provided on the Biology open website: https://journals.biologists.com/bio/pages/manuscript-prep#section-3-2

Please add (n = 9) and (n = 10) at the appropriate places on line 134.

Lines 141-146 are awkwardly separated into tiny paragraphs, merge these into one paragraph.

In Table 1, size is provided for each of the larvea and metamorphs. This needs to be changed to length presumably? I suggest this table be made more complete on the physical properties. It would be useful to have at least jaw length along the centro-sagittal plane, jaw width (at a specified place on the body, maybe something similar to a quadrate to quadrate measure?), and the weight of each the specimens should be included (if the weights are available/were taken - if not, these can be left out). The exact places of measurement need to be included in a figure (perhaps a supplementary figure) for ease of visualisation.

Can the authors be sure that feeding the metamorphs M1-4 vs M5-10 different meals will not lead to any differences between these two groups? A short justification beyond what has already been written about accustomed animals would be helpful in this respect.

The Phantom mentioned on line 154 was recording at 1000fps. The authors should state the resolution at which this was filmed. Please conjoin lines 156 and 157, there are too many tiny paragraphs in the article, which is disconcerting.

In Section 3, we need to see an illustration for the measurements of MGA, MG, MHA, and Mhd. Please include an illustration in either the methods section, or if you are at an in-article figure maximum, as an added supplementary element for easy reference. The methods section would need to be updated to this effect as well.

How was filming conducted to ensure a perfect or close to perfect lateral view of the animal, since this will affect the kinematic measurements and thus metrics? If the lateral view was not perfect, then there needs to be either a calibration method for the experimentation, or an error estimation method.

Line 189, SVL also needs to be illustrated and referred to, perhaps also as a supplementary figure.

Please write the test in 4.1 as a paragraph, not as a series of points.

In 4.2 please identify the independent and dependent variables in each two-tailed ANOVA test.

The authors use LMM's whenever an interaction term failed to impact a kinematic variable. It would be useful if the authors included the equation for the LMM, identifying the fixed effects and the random effects to make things easier for the reader to understand how the model is applied to the data.

Line 284, change explains to explain.

Figure 2(b) is the overall shade truly problematic such that it needs to be excluded? If yes, a deeper explanation would be helpful.

Figure 2, 3 and 4 - I suggest considering the application of a z-score to remove outliers as there are some very significantly visible outliers in places. This may also change the statistical output slightly.

The conclusions need to focus on the original hypothesis more effectively.

© 2026. Published by The Company of Biologists under the terms of the Creative Commons Attribution License (https://creativecommons.org/licenses/by/4.0/).

The supplementary material is very nice. While Figure 1 is generally informative, I felt I understood the kinematic much more through the videos. I suggest the authors take selected frames from any such videos that correspond with the images in Figure 1 (A and B, i, ii and iii) and add them under each as a set of sequence shots, as I believe it will improve the reader's perspective and understand of the Figure.

Reviewer 2

Comments for the author

I found the study interesting, and the question is relevant because the authors try to separate the effects of metamorphosis and body size on aquatic feeding kinematics. The system is also interesting because both larvae and metamorphs remain aquatic and use suction feeding. However, I think some parts of the manuscript need to be clarified, especially the conceptual framing, the statistical interpretation, and the strength of some conclusions.

Specific comments

1. The adaptive decoupling hypothesis is introduced as a major framework, but in some places the logic is not fully clear to me. The statement that metamorphosis remodels morphology and therefore may alter function is somewhat obvious. I think the authors should explain more clearly what the non-trivial prediction is here. In my view, the important prediction is not simply that larvae and metamorphs differ, but that the relationship between body size and feeding kinematics differs between developmental stages. I think this should be made clearer in the Introduction.

2. The manuscript says that ontogenetic decoupling has mostly been tested from a "phenotypic perspective". This wording is a bit vague because phenotype can include morphology, physiology, behaviour, and performance. From the context, the authors seem to mean mainly morphology. I suggest clarifying this by distinguishing morphological traits from functional performance.

3. Introduction states that metamorphosis is costly and that its widespread occurrence suggests that the benefits outweigh the costs. This is generally reasonable, but I think it may be too broad as written. Metamorphosis is very different across insects, amphibians, fishes, and other animals, so the costs may not be directly comparable across taxa. The authors could specify whether they are mainly referring to amphibian metamorphosis or to metamorphosis more generally.

4. The conclusion that the results suggest a morpho-functional trade-off is interesting, but I think it is currently stronger than what the data directly show. The study measures kinematic variables, but it does not directly measure suction force, water flow, prey escape risk, prey capture success, or cranial ossification in the studied individuals. Therefore, I think the authors should present this as a possible interpretation rather than as a solid, demonstrated trade-off.

5. The idea that larvae may benefit from labial lobes and gill slits is plausible and supported by previous literature, but in this study it is not directly tested. There is no manipulation of labial lobes or gill slits, and no measurement of pressure, flow, or prey escape. Therefore, I would like to suggest softening the wording throughout the Abstract and Discussion.

6. The authors suggest that metamorphs have faster movements because of increased cranial ossification. This is plausible, but ossification was not quantified in the individuals studied here. Therefore, the authors should make it clearer that this explanation is based on previous knowledge of metamorphic skull remodeling, not on direct measurements in this study.

7. The authors tested 9 larvae and 10 metamorphs, but four metamorphs correspond to individuals that were previously tested as larvae. These animals are treated as different individuals because they changed morphology and size. I think this needs further justification. Even after metamorphosis, these are still the same biological individuals (isn't it?), so they may not be fully independent statistically.

© 2026. Published by The Company of Biologists under the terms of the Creative Commons Attribution License (https://creativecommons.org/licenses/by/4.0/).

8. Another important point is that larvae and metamorphs occupy different size ranges (with no overlap). This makes it difficult to fully separate the effect of size from the effect of developmental stage. The size × developmental stage interaction is interesting, but the interpretation should be cautious as the stage-specific regressions are estimated over different size ranges.

9. The prey type was not the same for all animals. Larvae and some metamorphs were fed bloodworms, whereas other metamorphs were fed pieces of earthworm. Since prey type, size, or even material properties (specially the stiffness) can influence feeding kinematics, this should be discussed as a potential confounding factor. I assume the authors should explain why this difference is unlikely to affect the main results.

10. The authors used only successful feeding sequences in lateral view. This is understandable for kinematic analysis, but it means that the study cannot really draw strong conclusions about prey capture success or prey escape risk. This is important because the Discussion refers to prey escape risk. I suggest making this limitation explicit.

11. The authors state that when the interaction term was not significant, it was removed to increase statistical power. This approach should be justified more carefully. Removing terms based on significance can affect inference. I suggest reporting both the full and reduced models, or explaining that this model selection strategy was defined before analysis.

12. Theauthors transformed all variables using log1p to accommodate zero values. I think they should state which variables actually contained zero values and why these zeros are biologically meaningful. For some variables, such as time to maximum hyoid depression or maximum hyoid depression, zero values seem unusual in successful feeding sequences. This needs clarification.

13. Many kinematic variables were tested separately. The manuscript does not seem to address multiple testing. The authors should either apply a correction, justify why no correction was used, or consider a multivariate approach.

14. The Results section is quite brief and mostly descriptive. I think the authors should report more statistical information in the text, especially for the main results. For example, they could include key F-values, degrees of freedom, p-values, etc rather than relying entirely on Table 2 and the figures.

15. The interpretation of maximum hyoid depression is not clear. The authors first state that MG and Mhd are impacted by size, but then say that when the interaction is removed, the size effect for Mhd is no longer statistically significant. This should be rewritten.

16. The Abstract says that all duration variables increased with size in larvae and decreased with size in metamorphs. However, in Results some of these relationships are non-significant (or have I missed something?).

17. The authors suggest that larger gape likely allows larger prey capture. This is plausible, but prey size preference or prey capture performance was not directly tested (again, have I missed it?). I suggest keeping this interpretation cautious.

18. Figure 1 caption: The figure is useful, but the caption needs some language editing. For example, "followed quasi simultaneously" should be changed to "followed almost simultaneously", and "continuous expel of water" should be changed to "continuous expulsion of water". The figure would also benefit from clearer labels for structures such as gill slits, labial lobes, mouth opening, and hyoid movement.

19. Figure 2: For MG and Mhd, the figure shows a single regression line across larvae and metamorphs. Since the size ranges of larvae and metamorphs do not overlap, this line visually connects two separated groups and may imply a continuous relationship across a region with no data. I don't think that this was the intention here. The authors therefore should either acknowledge this, or consider another way to present the result.

© 2026. Published by The Company of Biologists under the terms of the Creative Commons Attribution License (https://creativecommons.org/licenses/by/4.0/).

20. Figure 2: Again here, the Mhd slope is positive but not statistically significant. The figure and text should not give the impression that Mhd clearly increases with size. The caption and Results should state that this is a non-significant trend.

21. Figure 3: it contains many panels, symbols, confidence bands, and slope/intercept values. The figure is informative but hard to read. I am not sure whether it would be possible to either splitting it into smaller figures?

22. Figure 3 and Figure 4: The raw points seem to represent feeding sequences, not individual means (am I right?). Since each individual contributes several sequences, the figures may visually inflate the sample size?

23. The axis labels such as "SVL log1p" and "TMG log1p" are not very clear.

24. Table 1:The column "Size" should be changed to "SVL (cm)" since SVL is the size variable used in the analyses. Is that the case?

25. The sentence "During metamorphosis, individuals can have difficulties feeding" perhaps better reads as "During metamorphosis, individuals may experience difficulty feeding."

Reviewer's Responses to Questions

Experimental quality
Are experiments performed using appropriate methods that will answer the question (or test the hypothesis or support the observations) posed by the authors? Is the right tool used for the job?
If 'No', please indicate reasons in Comments for Author box below.
Reviewer #1:

- Yes

Reviewer #2:

- Yes

Were the data analyzed using appropriate statistical tests?
If 'No', please indicate reasons in Comments for Author box below.
Reviewer #1:

- Yes

Reviewer #2:

- Yes

Reproducibility
Were experiments performed using adequate number of biological replicates?
If 'No', please indicate reasons in Comments for Author box below.
Reviewer #1:

- Yes

Reviewer #2:

- Yes

Is there sufficient raw data to assess the rigor of the analysis?
If 'No', please indicate reasons in Comments for Author box below.
Reviewer #1:

- Yes

Reviewer #2:

© 2026. Published by The Company of Biologists under the terms of the Creative Commons Attribution License (https://creativecommons.org/licenses/by/4.0/).

- Yes

Does the methods section provide sufficient detail to permit reproducibility?
If 'No', please indicate reasons in Comments for Author box below.
Reviewer #1:
- No

Reviewer #2:
- Yes

Completeness
Are the manuscript's conclusions supported by the data?
If 'No', please indicate reasons in Comments for Author box below.
Reviewer #1:
- No

Reviewer #2:
- No

Scholarship
Do the authors cite and discuss the merits of data that would argue for and against their conclusion?
If 'No', please indicate reasons in Comments for Author box below.
Reviewer #1:
- Yes

Reviewer #2:
- Yes

Does the manuscript title & abstract accurately reflect the contents of the manuscript, without hyperbole?
If 'No', please indicate reasons in Comments for Author box below.
Reviewer #1:
- Yes

Reviewer #2:
- Yes

First revision

<u>Author response to reviewers' comments</u>

General reply to the reviewers and editor:

We thank the reviewers for their positive and constructive comments, which have helped us improve the quality and clarity of our manuscript.

In response to their suggestions, we have notably revised the Introduction to provide additional context for the ontogenetic decoupling hypothesis. We have also restructured the final part of the Introduction so that the research gap, hypotheses, objectives, and predictions are now more clearly presented.

© 2026. Published by The Company of Biologists under the terms of the Creative Commons Attribution License (https://creativecommons.org/licenses/by/4.0/).

We have improved our statistical analyses by accounting for the second level of pseudoreplication that had been overlooked in the previous version as requested. We have also clarified the statistical design in the Materials and Methods section and, where appropriate, in the figure captions.

Finally, we have strengthened the Discussion by more explicitly acknowledging the limitations of our study and by adopting more cautious wording when interpreting our kinematic results. In particular, we now emphasize that these interpretations would benefit from complementary studies, such as direct measurements of prey capture success or intraoral pressure measurements.

These points summarize the main revisions made in response to the reviewer's comments. A detailed point-by-point response to each comment is provided below.

Reviewer 1:

I thoroughly enjoyed reading this article. The paper describes the use of a series of high-speed videos to characterise the kinematics of suction flow, specifically comparing larvae against metamorphs over a range of metrics. The work brings new information to the public domain and should be published after a revision that addresses the weaknesses. These are listed individually below:

The abstract is currently ca. 40 words over 200 words (see guidelines here: https://journals.biologists.com/bio/pages/manuscript-prep#section-3-2) - the authors would need to abridge some of the text.

OUR REPLY: We have abridged the abstract so that it now contains no more than 200 words, in accordance with the journal guidelines.

I did not see a summary statement (see here: https://journals.biologists.com/bio/pages/manuscript-prep#section-3-2) - though this might only be needed if the paper is accepted.

OUR REPLY: We have now added the summary statement as requested.

The authors have introduced the paper quite well, but I think there are some minor improvements needed here to broaden the readership value by improving on certain details. The authors state for example that, "The main hypotheses supporting this, is the 'adaptive' (or ontogenetic) decoupling hypothesis according to which, metamorphosis breaks the correlations among the phenotypic traits related to fitness expressed at the different life stages (Istock, 1967; Moran, 1994, Ten Brink et al., 2019)." - I would like the authors to provide some detail preceding this sentence to introduce the decoupling hypothesis more effectively, since the text transitions directly from a statement on the benefits of metamorphosis outweighing its costs. Could the authors provide a better transition by discussing this aspect in more detail (needs only a few sentences in my opinion, but flow is key and this transition is too abrupt). The decoupling hypothesis is again mentioned in line 126, so it is important that the reader fully understands what this hypothesis entails.

I find the text immediately after the same sentence reproduced above is also a little too presumptive in how it provides detail and context, i.e. straight after "The main hypotheses supporting this, is the 'adaptive' (or ontogenetic) decoupling hypothesis according to which, metamorphosis breaks the correlations among the phenotypic traits related to fitness expressed at the different life stages (Istock, 1967; Moran, 1994, Ten Brink et al., 2019)." in lines 51-56. I would like to suggest that the authors rewrite this section so that it (a) reads more clearly and (b) provides a little further detail on things like "each stage" and how exactly post-metamorphic life stages can develop independently from pre-metamorphic stages leading to reduced "inter-stage competition". This section feels like it needs better flow and explanation as it the way it currently reads, doesn't lead the reader in any particular direction as these terminologies are very generic. In some ways a lot of this is easier to understand in the proceeding paragraph.

© 2026. Published by The Company of Biologists under the terms of the Creative Commons Attribution License (https://creativecommons.org/licenses/by/4.0/).

OUR REPLY: We thank the reviewer for this constructive comment. We agree that the transition between the statement that the prevalence of metamorphosis suggests that its benefits may outweigh its costs and the introduction of the adaptive (or ontogenetic) decoupling hypothesis was too abrupt. We have therefore smoothened the transition by adding more context on the different selective pressures that individuals experience throughout ontogeny (L 48-59). This additional information provides a clearer rationale for why metamorphosis may be beneficial and allows us to introduce the adaptive decoupling hypothesis more gradually. We now better explain how metamorphosis may reduce inter-stage competition. In particular, we now clarify how the acquisition of distinct phenotypes at different life stages can allow pre- and post-metamorphic individuals to exploit different food resources and adapt to their respective ecological and physiological demands (L 54-59).

The proceeding paragraph is very good but the "partial decoupling" mentioned on line 80 is treated again, somewhat too frivolously, so I would ask the authors to clarify what a partial decoupling entails here in better detail. These details in the introductory text are important, as the set the scene for the rest of the paper and the introduction "almost" does that but not quite effectively enough.

OUR REPLY: We have revised the manuscript to clarify what is meant by "partial decoupling" as referred to by Johansson et al. (2010). We now explicitly explain that larval swimming and juvenile jumping performance are decoupled, but remain indirectly linked through body size effects (L87-91).

The last two paragraphs of the introduction need to be reordered more sensibly. The first of the two starts with aims (line 103) and then there is a paragraph (starting line 124) that is two lines long describing expectations. Please rearrange this such that there is only one paragraph, the research gap is properly identified (leading in from the details provided in preceding paragraphs and details available within these paragraphs), the hypothesis of the authors' paper is stipulated clearly (merge the expectations into this perhaps?), followed by the aims of the present paper. This can then be followed by text stipulating how (generally) the authors will enable these aims and how they expect to be able thence, to accept or reject their hypothesis/expectations.

OUR REPLY: We have restructured the final part of the Introduction to improve logical flow and clarity. We now first explicitly identify the research gap, before stating that we expect both developmental stage and body size to have an effect on feeding kinematics. We then summarize the objective of our study and describe specifically what we test. We conclude this section by clearly stating that, if both body size and developmental stage influence feeding kinematics as expected, this should be reflected in kinematic variables scaling differently with body size across developmental stages.

Biology Open as far as I know, usually places the results immediately after the introduction, followed by discussion and the methods are at the end. The results therefore need to be written in a way that flows from an introduction and the authors should format/revise the textual flow of the results section in order to accommodate this. These details are provided on the Biology open website: https://journals.biologists.com/bio/pages/manuscript-prep#section-3-2

OUR REPLY: We have now revised the manuscript to comply with the *Biology Open* format, in which the Results section follows the Introduction and is followed by the Discussion, with the Materials and Methods placed at the end of the manuscript. Accordingly, we have reorganized the manuscript structure and adjusted the flow of the Results section to ensure a coherent progression from the Introduction to the Results, as recommended in the journal guidelines.

Please add (n = 9) and (n = 10) at the appropriate places on line 134.

OUR REPLY: We have added (n=9) and (n=10) as suggested (L362-363).

Lines 141-146 are awkwardly separated into tiny paragraphs, merge these into one paragraph.

OUR REPLY: We have merged these points into one paragraph as suggested (now L362-371)

© 2026. Published by The Company of Biologists under the terms of the Creative Commons Attribution License (https://creativecommons.org/licenses/by/4.0/).

In Table 1, size is provided for each of the larvae and metamorphs. This needs to be changed to length presumably? I suggest this table be made more complete on the physical properties. It would be useful to have at least jaw length along the centro-sagittal plane, jaw width (at a specified place on the body, maybe something similar to a quadrate to quadrate measure?), and the weight of each the specimens should be included (if the weights are available/were taken - if not, these can be left out). The exact places of measurement need to be included in a figure (perhaps a supplementary figure) for ease of visualisation.

OUR REPLY: We have revised “size” to snout-vent length in the table (now Table 2) and have added a supplementary figure illustrating the measurement protocol, as suggested (Fig. S2). We did not include jaw length, jaw width, or body mass, as these variables were not used in our statistical design and including them would not further contribute to the aims of the study.

Can the authors be sure that feeding the metamorphs M1-4 vs M5-10 different meals will not lead to any differences between these two groups? A short justification beyond what has already been written about accustomed animals would be helpful in this respect.

OUR REPLY: Please see our response to Reviewer 2, Comment 9, where we discuss this limitation in detail and explain why we consider it unlikely to affect our main conclusions.

The Phantom mentioned on line 154 was recording at 1000fps. The authors should state the resolution at which this was filmed.

OUR REPLY: We have added this information as requested

Please conjoin lines 156 and 157, there are too many tiny paragraphs in the article, which is disconcerting.

OUR REPLY: We have joined the lines as suggested (now L 380-383)

In Section 3, we need to see an illustration for the measurements of MGA, MG, MHA, and Mhd. Please include an illustration in either the methods section, or if you are at an in-article figure maximum, as an added supplementary element for easy reference. The methods section would need to be updated to this effect as well.

OUR REPLY: We have added a supplementary figure (Fig. S2B) illustrating the measurement of MGA, MG, MHA, and Mhd as suggested.

How was filming conducted to ensure a perfect or close to perfect lateral view of the animal, since this will affect the kinematic measurements and thus metrics? If the lateral view was not perfect, then there needs to be either a calibration method for the experimentation, or an error estimation method.

OUR REPLY: We only retained sequences in which head length measured from the lateral-view videos deviated by less than 3% from the actual head length of the animal. Consequently, we had to discard a significant number of videos from the analyses, but this ensured that only recordings in lateral view were included.

Line 189, SVL also needs to be illustrated and referred to, perhaps also as a supplementary figure.

OUR REPLY: We have added a supplementary figure to illustrate how SVL was measured (Fig. S2A).

Please write the test in 4.1 as a paragraph, not as a series of points.

OUR REPLY: We have rewritten the test in Section 4.1 as a single paragraph, as suggested.

In 4.2 please identify the independent and dependent variables in each two-tailed ANOVA test.

© 2026. Published by The Company of Biologists under the terms of the Creative Commons Attribution License (https://creativecommons.org/licenses/by/4.0/).

OUR REPLY: We have added, in parentheses, the corresponding explanatory (independent) and dependent variables for the two-tailed ANOVA test (L 438).

The authors use LMM's whenever an interaction term failed to impact a kinematic variable. It would be useful if the authors included the equation for the LMM, identifying the fixed effects and the random effects to make things easier for the reader to understand how the model is applied to the data.

OUR REPLY: Following the reviewer's suggestion, we have now explicitly specified in the text the fixed and random effects included in the linear mixed-effects models (L422-427). We have also added the corresponding R formula syntax (L430-431 and L442-443) to clearly illustrate the model structure and improve the readability and interpretation of the statistical analyses.

Line 284, change explains to explain.

OUR REPLY: We have corrected the typographical error (now line 226).

Figure 2(b) is the overall shade truly problematic such that it needs to be excluded? If yes, a deeper explanation would be helpful.

OUR REPLY: The difference in the graphical representation is determined by the significance of the interaction term in the Type II ANOVA results.

When the interaction between developmental stage and size is significant, as for maximum gape angle (MGA; Figure 2B), it indicates that the effect of size differs between larvae and metamorphs. In this case, separate regression lines are fitted for each developmental stage, each with its own confidence interval (shaded area), because the relationship between size and the kinematic variable is stage-dependent.

In contrast, when the interaction term is not significant and only size has a significant effect, as for maximum gape distance (MG; Figure 2A), a single regression line is fitted across all individuals because the relationship between size and the kinematic variable does not differ between developmental stages. Accordingly, a single overall confidence interval is displayed.

Therefore, no overall shaded area was excluded from Figure 2B. Rather, the statistical model supports separate regressions for each developmental stage instead of a single common regression. We have revised the figure caption to explain this distinction more clearly.

Figure 2, 3 and 4 - I suggest considering the application of a z-score to remove outliers as there are some very significantly visible outliers in places. This may also change the statistical output slightly.

OUR REPLY: We identified one video sequence ("Ind3, Video 3" from metamorph M1) in which the individual exhibited an atypical feeding behaviour. During this sequence, the hyoid was not depressed, resulting in values of zero for maximum hyoid depression (Mhd), time to maximum hyoid depression (TMhd), and hyoid elevation duration (DhdE). Because this behaviour is not representative of normal suction feeding, we removed this sequence from the dataset prior to the analyses.

Apart from the exclusion of this atypical sequence, we did not apply a z-score to remove outliers. We consider the remaining extreme values to reflect genuine biological variation in prey capture performance rather than measurement or tracking errors. Removing these points could therefore exclude biologically meaningful variation and potentially bias the interpretation of the results

The conclusions need to focus on the original hypothesis more effectively.

OUR REPLY: Following the revision of the Introduction and the addition of a sentence (L333-335) explicitly relating our main findings to the hypothesis stated at the end of the Introduction (L139-141), the Conclusions now focus more effectively on the original hypothesis.

© 2026. Published by The Company of Biologists under the terms of the Creative Commons Attribution License (https://creativecommons.org/licenses/by/4.0/).

The supplementary material is very nice. While Figure 1 is generally informative, I felt I understood the kinematic much more through the videos. I suggest the authors take selected frames from any such videos that correspond with the images in Figure 1 (A and B, i, ii and iii) and add them under each as a set of sequence shots, as I believe it will improve the reader's perspective and understand of the Figure.

OUR REPLY: We have revised Figure 1 by adding representative frames from the supplementary videos corresponding to each schematic illustration (A.i–iv and B.i–iv).

Reviewer 2: General comments

I found the study interesting, and the question is relevant because the authors try to separate the effects of metamorphosis and body size on aquatic feeding kinematics. The system is also interesting because both larvae and metamorphs remain aquatic and use suction feeding. However, I think some parts of the manuscript need to be clarified, especially the conceptual framing, the statistical interpretation, and the strength of some conclusions.

Specific comments

1. The adaptive decoupling hypothesis is introduced as a major framework, but in some places the logic is not fully clear to me. The statement that metamorphosis remodels morphology and therefore may alter function is somewhat obvious. I think the authors should explain more clearly what the non-trivial prediction is here. In my view, the important prediction is not simply that larvae and metamorphs differ, but that the relationship between body size and feeding kinematics differs between developmental stages. I think this should be made clearer in the Introduction.

OUR REPLY: The important prediction is indeed that the relationship between body size and feeding kinematics differs between developmental stages. This point was already included in the previous version of the manuscript, where we stated that kinematic variables may scale differently with size depending on developmental stage. However, this may not have been sufficiently clear in the overall flow of the Introduction. Therefore, following Reviewer 1's suggestion, we have reorganized the final part of the Introduction to make this prediction more explicit. We now conclude the introduction with this statement, as it clearly defines the expected pattern of results that will allow us to evaluate our hypothesis that both body size and developmental stage influence feeding kinematics.

2. The manuscript says that ontogenetic decoupling has mostly been tested from a "phenotypic perspective". This wording is a bit vague because phenotype can include morphology, physiology, behaviour, and performance. From the context, the authors seem to mean mainly morphology. I suggest clarifying this by distinguishing morphological traits from functional performance.

OUR REPLY: We have replaced the term "phenotypic perspective" with "morphological perspective" to clarify that we refer to morphological traits in this sentence.

3. Introduction states that metamorphosis is costly and that its widespread occurrence suggests that the benefits outweigh the costs. This is generally reasonable, but I think it may be too broad as written. Metamorphosis is very different across insects, amphibians, fishes, and other animals, so the costs may not be directly comparable across taxa. The authors could specify whether they are mainly referring to amphibian metamorphosis or to metamorphosis more generally.

OUR REPLY: We agree that metamorphosis varies considerably among taxa, including insects, amphibians, fishes, and other animals, and that the costs and benefits of metamorphosis may differ depending on the specific developmental context. However, because metamorphosis is a widespread phenomenon across both vertebrates and invertebrates, and because the adaptive (ontogenetic) decoupling hypothesis provides a general framework applicable to all types of organisms undergoing metamorphosis, we intentionally introduced this concept broadly rather than restricting it to amphibians. Nevertheless, the transition from the general statement that

© 2026. Published by The Company of Biologists under the terms of the Creative Commons Attribution License (https://creativecommons.org/licenses/by/4.0/).

the benefits of metamorphosis may outweigh its costs to the adaptive decoupling hypothesis was too abrupt, which may have made the scope of the argument unclear. We have therefore revised this section by adding more context on how metamorphosis can reduce inter-stage competition. This revised explanation still refers to metamorphosis as a broad evolutionary phenomenon, while providing a clearer rationale for the relevance of the adaptive decoupling hypothesis to our study.

4. The conclusion that the results suggest a morpho-functional trade-off is interesting, but I think it is currently stronger than what the data directly show. The study measures kinematic variables, but it does not directly measure suction force, water flow, prey escape risk, prey capture success, or cranial ossification in the studied individuals. Therefore, I think the authors should present this as a possible interpretation rather than as a solid, demonstrated trade-off.

OUR REPLY: We have expanded the conclusion to highlight that future studies should combine kinematic analyses with measurements of cranial ossification, prey capture success, and intraoral pressure across an ontogenetic series to directly test this proposed morpho-functional trade-off (L 348-351).

5. The idea that larvae may benefit from labial lobes and gill slits is plausible and supported by previous literature, but in this study, it is not directly tested. There is no manipulation of labial lobes or gill slits, and no measurement of pressure, flow, or prey escape. Therefore, I would like to suggest softening the wording throughout the Abstract and Discussion.

OUR REPLY: To address this point, we have expanded the paragraph (Lines 295-301). This paragraph already acknowledged that further analyses are required to test whether the proposed morpho-functional trade-off contributes to reducing interstage competition. We have now clarified that additional analyses are needed to determine whether the morpho-functional trade-off inferred here actually exists and whether it contributes to reducing interstage competition. As one possible approach, we proposed comparing prey capture success between larvae and metamorphs. We have also added that measurements of intraoral pressure would be necessary to directly test this hypothesis. Finally, we have reiterated this perspective in the conclusion (Lines 348-351).

6. The authors suggest that metamorphs have faster movements because of increased cranial ossification. This is plausible, but ossification was not quantified in the individuals studied here. Therefore, the authors should make it clearer that this explanation is based on previous knowledge of metamorphic skull remodeling, not on direct measurements in this study.

OUR REPLY: To address this point, we have revised the discussion to make it explicit that cranial ossification was not quantified in the present study and that our proposed explanation is based on previous work describing skull remodelling during metamorphosis (Lines 214-215).

7. The authors tested 9 larvae and 10 metamorphs, but four metamorphs correspond to individuals that were previously tested as larvae. These animals are treated as different individuals because they changed morphology and size. I think this needs further justification. Even after metamorphosis, these are still the same biological individuals (isn't it?), so they may not be fully independent statistically.

OUR REPLY: We agree with the reviewer that the four metamorphs corresponding to previously tested larvae are not statistically independent because they represent repeated measurements of the same biological individuals. This was not adequately accounted for in the previous version of the manuscript.

To address this issue, we assigned two identifiers to each feeding sequence. The first identifier (stage_ID; L1–L9 for larvae and M1–M10 for metamorphs) corresponds to the individual at a given developmental stage. The second identifier (ind_biol; ind1–ind15) represents the biological identity of each animal regardless of developmental stage, allowing us to link the four individuals that were measured both as larvae and as metamorphs.

© 2026. Published by The Company of Biologists under the terms of the Creative Commons Attribution License (https://creativecommons.org/licenses/by/4.0/).

Our linear mixed-effects models now include both random effects, (1 | stage-specific identifier) and (1 | biological identifier). The first accounts for the non-independence of repeated feeding sequences recorded from the same individual within a developmental stage (pseudoreplication), whereas the second accounts for the repeated measurements of the same biological individuals across developmental stages. This is now incorporated in lines 421-434 of the Materials and Methods subsection entitled "4.1 Statistical modelling of kinematic variables".

Notice that including only (1 | biological identifier) would not adequately represent the structure of our data because the repeated feeding sequences recorded from a given individual within a developmental stage are expected to be more similar to one another than to sequences recorded from the same individual after metamorphosis, when morphology and body size have changed substantially. The revised model therefore accounts for both sources of non-independence.

8. Another important point is that larvae and metamorphs occupy different size ranges (with no overlap). This makes it difficult to fully separate the effect of size from the effect of developmental stage. The size × developmental stage interaction is interesting, but the interpretation should be cautious as the stage-specific regressions are estimated over different size ranges.

OUR REPLY: We agree that an overlap in body size between larvae and metamorphs would have facilitated a more direct comparison of the effects of developmental stage and body size. However, the absence of size overlap does not prevent the linear mixed-effects models from estimating the independent effects of developmental stage, body size, and their interaction. These effects are estimated simultaneously using the variation in body size observed within each developmental stage. Furthermore, the fact that maximum gape (MG) was influenced only by body size, whereas most other variables showed a significant size × developmental stage interaction, supports the ability of the models to distinguish between the effects of body size and developmental stage. Nevertheless, we agree that the lack of overlap in body size may limit the strength of the inferences that can be drawn from the regressions. We have therefore mentioned this limitation when interpreting the MG results in the Discussion. In addition, we have retained cautious wording throughout the Discussion, using conditional language (e.g. *may*) when interpreting these findings.

9. The prey type was not the same for all animals. Larvae and some metamorphs were fed bloodworms, whereas other metamorphs were fed pieces of earthworm. Since prey type, size, or even material properties (specially the stiffness) can influence feeding kinematics, this should be discussed as a potential confounding factor. I assume the authors should explain why this difference is unlikely to affect the main results.

OUR REPLY: We agree that using different prey types for larvae and some metamorphs versus other metamorphs is a limitation of our study. The main difference between the two prey types is their size, particularly their transverse diameter. The variables most likely to be affected by prey size are those related to buccal expansion, namely maximal gape distance (MG), maximal gape angle (MGA), and maximal hyoid depression (Mhd).

However, several observations suggest that prey size is unlikely to explain the patterns observed for MG and Mhd. First, maximal gape is well described by a single regression across larvae and metamorphs, with no evidence of a change in slope between groups (Fig. 3A). This suggests that salamander body size has a much stronger influence on MG than prey size. Second, if larger prey were driving the pattern observed for Mhd, we would expect metamorphs feeding on earthworm pieces to exhibit greater Mhd values than those feeding on bloodworms. Inspection of the raw data (Fig. 2C) does not support this expectation. If anything, most sequences from metamorphs fed bloodworms exhibit greater Mhd values than those from metamorphs fed earthworm pieces.

Nevertheless, we acknowledge that prey size may have influenced maximum gape angle. In particular, the break in slope observed could reflect either a change in the relationship of the variable with size following metamorphosis, or either reflect the difference of prey offered with smaller prey offered to metamorphs M1, M2, M3 and M5 and bigger prey offered to

© 2026. Published by The Company of Biologists under the terms of the Creative Commons Attribution License (https://creativecommons.org/licenses/by/4.0/).

metamorphs M4, M6-M10. Consequently, we cannot completely exclude a modest effect of prey size on this variable. Nevertheless, any such effect appears limited, as no comparable discontinuity is observed for MG, which would also be expected to respond to prey size (Fig. 3A). We have now explicitly acknowledged this limitation in the manuscript (Lines 174 and 185).

Finally, prey size is unlikely to account for the observed differences in temporal variables. If prey size were the dominant factor, larger prey would be expected to increase feeding duration. In contrast, we observed the opposite pattern. Furthermore, because MG-one of the variables expected to be most sensitive to prey size-shows no evidence of a prey-type effect, we are confident that the reported differences in feeding durations primarily reflect the explanatory variables investigated rather than prey characteristics.

10. The authors used only successful feeding sequences in lateral view. This is understandable for kinematic analysis, but it means that the study cannot really draw strong conclusions about prey capture success or prey escape risk. This is important because the Discussion refers to prey escape risk. I suggest making this limitation explicit.

OUR REPLY: We agree that our data do not allow direct conclusions about prey capture success or prey escape risk. Our interpretations were already formulated cautiously using terms such as *may*. To make this limitation more explicit, we have now added statements in the Discussion emphasizing that further studies, including analyses of unsuccessful feeding attempts, will be necessary to test these hypotheses. These additions have been made in the subsection *"Reduced interstage competition remains to be confirmed"* (L295-301) and at the end of the General Conclusions (L348-351).

11. The authors state that when the interaction term was not significant, it was removed to increase statistical power. This approach should be justified more carefully. Removing terms based on significance can affect inference. I suggest reporting both the full and reduced models, or explaining that this model selection strategy was defined before analysis.

OUR REPLY: Only maximum gape distance (MG) and maximum hyoid depression (Mhd) were not impacted by the interaction term. For these variables, we compared the full model (including the interaction term) with the reduced model (without the interaction term) using the Akaike and Bayesian Information Criteria (AIC and BIC respectively) to evaluate model fit. In both cases, the reduced model provided a better fit to the data, as indicated by lower AIC and BIC values, which are now reported in the manuscript and in table 1. We have clarified this model selection procedure in the Materials and Methods section by explicitly stating that AIC and BIC comparisons were used to determine whether the interaction term should be retained.

For MG and Mhd, the predicted regression lines shown in the figures are therefore based on the reduced models. However, the parameter estimates for both the full and reduced models are provided in the revised Supplementary Table S2, and the ANOVA results for both the full and reduced models are reported in the revised Table 1. These tables were already included in the previous version of the manuscript, but they have been updated following the revision of our linear mixed models. In addition, we have now included the QQ plots for the full models of MG and Mhd in Figure S2, which were missing from the previous version.

12. The authors transformed all variables using log1p to accommodate zero values. I think they should state which variables actually contained zero values and why these zeros are biologically meaningful. For some variables, such as time to maximum hyoid depression or maximum hyoid depression, zero values seem unusual in successful feeding sequences. This needs clarification.

OUR REPLY: In our original dataset, zero values occurred for three variables - maximum hyoid depression (Mhd), time to maximum hyoid depression (TMhd), and duration of hyoid elevation (DhdE) - in a single feeding sequence (sequence "indiv n3 vid 3"; row 36 of Table S1) from metamorph M1. These zero values resulted from an atypical feeding event during which the salamander did not depress its hyoid. Because this behaviour is highly unusual, we decided to exclude this sequence from the dataset. Consequently, log1p transformation was no longer

© 2026. Published by The Company of Biologists under the terms of the Creative Commons Attribution License (https://creativecommons.org/licenses/by/4.0/).

required for these variables, the statistical analyses were updated accordingly, and only maximum head angle (MHA), which includes a genuine zero value, remains log1p-transformed.

13. Many kinematic variables were tested separately. The manuscript does not seem to address multiple testing. The authors should either apply a correction, justify why no correction was used, or consider a multivariate approach.

OUR REPLY: Our approach consists of decomposing the suction-feeding sequence into distinct functional components. Our objective was not to determine whether feeding kinematics differ globally among developmental stages or as a function of body size, which would indeed be more appropriately addressed using a multivariate approach. Rather, we aimed to identify which specific phases of the suction-feeding sequence are influenced by body size, developmental stage, and their interaction.

Accordingly, each kinematic variable was analysed separately because it quantifies a distinct biomechanical event occurring during a specific phase of suction feeding (e.g., buccal expansion, jaw closing, or hyoid elevation), rather than representing repeated measurements of a single underlying response.

The only exceptions are gape duration (DG) and prey capture duration (PCD), which are composite variables derived from the constituent kinematic variables. These composite variables were retained primarily to facilitate comparisons with previous studies on salamander feeding kinematics, in which they are commonly reported. However, our biological interpretation relies primarily on their constituent variables because these decompose the suction-feeding sequence into functionally meaningful phases and therefore provide greater insight into how body size and developmental stage affect each step of the feeding mechanism.

Finally, the mixed-effects models constitute the primary inferential framework of our study, as they estimate the effects of body size, developmental stage, and their interaction while accounting for the hierarchical structure of the data through random effects. The ANOVA tables were included to summarize the significance of the fixed effects estimated by the fitted mixed-effects models, but our conclusions rely primarily on the estimated effects and their predicted relationships shown in the figures, rather than solely on statistical significance.

For these reasons, we do not consider a global multiple-testing correction to be appropriate for our study.

14. The Results section is quite brief and mostly descriptive. I think the authors should report more statistical information in the text, especially for the main results. For example, they could include key F-values, degrees of freedom, p-values, etc rather than relying entirely on Table 2 and the figures.

OUR REPLY: We have intentionally kept the Results section concise and focused on the main biological patterns rather than reporting all statistical outputs in the text. Indeed, our analyses include ten kinematic variables, and providing F-values, degrees of freedom, and p-values for each variable would substantially increase the length of the section and could obscure the main findings, particularly for variables showing similar patterns. We have therefore retained the complete statistical information in the tables and figures, while using the text to highlight the major trends and overall patterns observed in the data. We believe that this structure improves readability and allows readers to more easily identify the key results.

15. The interpretation of maximum hyoid depression is not clear. The authors first state that MG and Mhd are impacted by size, but then say that when the interaction is removed, the size effect for Mhd is no longer statistically significant. This should be rewritten.

OUR REPLY: Following revision of the linear mixed-effects models and the exclusion of one atypical feeding sequence in which a metamorph (M1) did not depress its hyoid, the results for maximum hyoid depression (Mhd) have changed. In the revised analysis, Mhd is significantly affected by both size and developmental stage in the full model (including the interaction

© 2026. Published by The Company of Biologists under the terms of the Creative Commons Attribution License (https://creativecommons.org/licenses/by/4.0/).

term), whereas only developmental stage remains significant in the reduced model (without the interaction term). We have revised the Results and Discussion sections accordingly.

16. The Abstract says that all duration variables increased with size in larvae and decreased with size in metamorphs. However, in Results some of these relationships are non-significant (or have I missed something?).

OUR REPLY: We have revised the Abstract to accurately reflect our results. It now states that “most”, rather than “all”, duration variables increased with size in larvae and decreased with size in metamorphs.

17. The authors suggest that larger gape likely allows larger prey capture. This is plausible, but prey size preference or prey capture performance was not directly tested (again, have I missed it?). I suggest keeping this interpretation cautious.

OUR REPLY: We have made this limitation more explicit by acknowledging that future studies investigating prey capture performance across a range of prey sizes and evaluating prey size preferences in larvae and metamorphs would provide a more direct test of this hypothesis (L202-205)

18. Figure 1 caption: The figure is useful, but the caption needs some language editing. For example, "followed quasi simultaneously" should be changed to "followed almost simultaneously", and "continuous expel of water" should be changed to "continuous expulsion of water". The figure would also benefit from clearer labels for structures such as gill slits, labial lobes, mouth opening, and hyoid movement.

OUR REPLY: We have made the suggested language revisions in the Figure 1 caption. In addition, we have added labels identifying key anatomical structures, including the gills, gill slit, and labial lobe. To further improve the clarity of the figure, we have numbered the red arrows indicating the successive jaw and hyoid movements. These numbered movements are now clearly defined in the figure legend, making the arrows easier to interpret.

19. Figure 2: For MG and Mhd, the figure shows a single regression line across larvae and metamorphs. Since the size ranges of larvae and metamorphs do not overlap, this line visually connects two separated groups and may imply a continuous relationship across a region with no data. I don't think that this was the intention here. The authors therefore should either acknowledge this, or consider another way to present the result.

OUR REPLY: We intentionally represented a single regression line for maximum gape distance (MG; Figure 2A). Indeed, because the interaction term was not significant and only size had a significant effect, a single regression line was fitted across all individuals. This representation reflects the ANOVA results and directly visualizes the predictions of the second linear mixed-effects model (without the interaction term), indicating that the relationship between size and the kinematic variable does not differ between developmental stages.

We acknowledge that the results could potentially have differed if the size ranges of larvae and metamorphs had overlapped, and we now mention this limitation in the Discussion (L173-174). However, we have chosen to retain the single regression line because it directly visualizes the relationship predicted by the model. In addition, we have clarified in the figure caption that a single regression line is shown because only size had a significant effect.

We also note that, following the revised analyses, the results for maximum hyoid depression (Mhd) have changed, and the figure has been updated accordingly.

20. Figure 2: Again here, the Mhd slope is positive but not statistically significant. The figure and text should not give the impression that Mhd clearly increases with size. The caption and Results should state that this is a non-significant trend.

OUR REPLY: Following the revised analyses and the removal of the video sequence in which metamorph M1 exhibited atypical feeding behaviour (i.e., no hyoid depression during the

© 2026. Published by The Company of Biologists under the terms of the Creative Commons Attribution License (https://creativecommons.org/licenses/by/4.0/).

feeding sequence), the results for maximum hyoid depression (Mhd) have changed. Mhd is now affected only by developmental stage, resulting in separate regression lines for larvae and metamorphs. Although neither regression line shows a significant relationship with size, the graphical representation illustrates that Mhd is generally higher in metamorphs than in larvae, reflecting the significant effect of developmental stage.

The original figure caption already indicated that statistically significant slopes were denoted by an asterisk (*). To make the absence of a significant relationship more explicit, we now also indicate non-significant slopes with the label "NS" in the figure. This has been clarified in the caption. We have also revised the Results and Discussion accordingly to reflect these updated analyses.

21. Figure 3: it contains many panels, symbols, confidence bands, and slope/intercept values. The figure is informative but hard to read. I am not sure whether it would be possible to either splitting it into smaller figures?

OUR REPLY: We have split the original Figure 3 into two separate figures to improve readability as suggested. The first figure now presents only mouth movements (formerly panel A), whereas the second presents hyoid movements together with prey capture duration (formerly panels B and C). Although prey capture duration (PCD) represents the overall duration of the feeding event, we chose to keep it with the hyoid variables because DhdE was calculated as PCD − TMhd, making these variables closely related.

22. Figure 3 and Figure 4: The raw points seem to represent feeding sequences, not individual means (am I right?). Since each individual contributes several sequences, the figures may visually inflate the sample size?

OUR REPLY: The graphs combine two complementary types of information by superimposing the raw data and the predictions from the linear mixed-effects models. The raw data points shown in Figures 2, 3, and 4 indeed represent individual feeding sequences rather than individual means. Displaying these points allows readers to visualize the distribution and variability of the observed data and to assess whether the predicted regression lines are consistent with the raw observations. The regression lines themselves are predictions from the linear mixed-effects models (i.e. their slopes correspond to the model estimates). Consequently, they account for the non-independence of repeated feeding sequences from the same individual, as well as the correlation introduced by the four individuals that were filmed at both developmental stages.

To avoid any ambiguity, we have clarified in the Materials and Methods (L462–464) and in the figure captions (Fig. 2-5) that the graphs display both the raw feeding sequences and the predictions from the linear mixed-effects models.

23. The axis labels such as "SVL log1p" and "TMG log1p" are not very clear.

OUR REPLY: In the revised manuscript, we have reconsidered the data transformations and now use a log10 transformation instead of a log1p transformation for all variables, except maximum head angle (MHA; Fig. 5), for which a log1p transformation was retained because the dataset includes a zero value. Accordingly, only the axis labels "SVL log1p" and "MHA log1p" remain, but we now explicitly explain in the figure caption that log1p corresponds to the natural logarithm of *(1 + x)* (Fig. 5).

24. Table 1: The column "Size" should be changed to "SVL (cm)" since SVL is the size variable used in the analyses. Is that the case?

OUR REPLY: We have incorporated the suggested correction in the table (Now table 2)

25. The sentence "During metamorphosis, individuals can have difficulties feeding" perhaps better reads as "During metamorphosis, individuals may experience difficulty feeding."

OUR REPLY: We have incorporated the suggested correction.

© 2026. Published by The Company of Biologists under the terms of the Creative Commons Attribution License (https://creativecommons.org/licenses/by/4.0/).

Second decision letter

MS ID#: bio.062752R1

MS Title: The impact of metamorphosis and size on the aquatic feeding kinematics of the Iberian ribbed newt (Pleurodeles waltl Michahelles, 1830)

Authors: Isabelle Toussaint-Lardé; Merlin Hugon-Daune-Fäs; Anthony Herrel; Anne-Claire Fabre

I have now reached a decision on the above manuscript. Both the reviewers and I agree that the authors did well in addressing the major comments. Reviewer 2 pointed out some minor inconsistencies and errors that require corrections before I can accept the paper. I also agree with Reviewer 1 to put the data (including videos) in an online repository instead of upon request. Please check thoroughly for any grammatical, typological, and spelling errors as they can lead to misinterpretations of the study.

At this stage, we also ask you to ensure your manuscript complies with our formatting guidelines – please see our manuscript preparation guidelines for details. Provided you are able to fully address the referees' comments, we are positive about publication of your paper (we accept over 95% of revision submissions) and therefore hope you won't mind any extra work involved in reformatting your manuscript at this point.

Please upload both a 'clean' version of your Word file, along with a highlighted version clearly showing where you have made changes in the revised manuscript. Please avoid using 'Track changes' in Word files as these are lost in PDF conversion.

I should be grateful if you would also provide a point-by-point response detailing how you have dealt with the points raised by the reviewers in the 'Response to Reviewers' box. Please attend to all of the reviewers' comments. If you do not agree with any of their criticisms or suggestions please explain clearly why this is so.

Reviewer 1

Comments for the author

I have read the responses to review, and the revised version of the manuscript. I am satisfied with the authors' revisions to this article. The readbility and flow has been significantly improved and I feel the results and particularly the discussion, provide novel insights that are of value to the community. There is only one minor issue that remains. The authors declare that videos will be made available on request. The videos are actually the raw datasets for this paper, and as such, would be better made openly available at the time of publication. I would like to ask the authors to upload the videos into an online data repository and to provide the link to the raw video datasets under Data Availability, so that they are accessible to the readers.

Reviewer 2

Comments for the author

I appreciate the author's efforts in addressing my previous comments. The revised manuscript is substantially improved, and the authors have addressed most of my main concerns. In particular, I am pleased to see that the Discussion is now more cautious and now acknowledges some limitations.

© 2026. Published by The Company of Biologists under the terms of the Creative Commons Attribution License (https://creativecommons.org/licenses/by/4.0/).

I nevertheless think that some issues remain before the manuscript is ready for publication. I am not suggesting any new experiments, but I think that the points below should be addressed prior to the pub,lication. There are so many small consistencies in reporting of the values. I could not list all of them, but they are critical and should be fixed.

Comment 1: Larvae and metamorphs still occupy completely non-overlapping SVL ranges. The models can compare the scaling relationships within each developmental stage, but this does not mean that developmental-stage effects are fully independent of body size. I believe that statements such as metamorphs having greater hyoid depression "independent of body size" should be a little bit softened. The lack of size overlap should also be acknowledged as a limitation of the stage comparisons, rather than only in relation to maximum gape.

Comment 2: The revised manuscript now acknowledges that larvae and some metamorphs were fed bloodworms, whereas other metamorphs were fed pieces of earthworm. However, the inspection of the raw patterns cannot exclude the possible effect of prey size, type, or stiffness on the observed kinematics. A sensitivity analysis would be useful (provided that the remaining sample is adequate?). Otherwise, the authors would need to avoid suggesting that prey effects have been ruled out or should add commentaries.

Comment 3: The captions of Figs 3-5 incorrectly state that a significant slope has a 95% confidence interval "above zero." I can see that some significant slopes are negative. Therefore this should be changed to "the 95% confidence interval excludes zero."

Comment 4: The numerical values reported in the text should be checked very carefully against those shown in the figures. For example, the MGA slope values and the reported DhdE slope ratio appear to be inconsistent across different parts of the manuscript. I could not list all of them but an example is where Discussion reports slopes of 0.28 for larvae and 2.28 for metamorphs, but Fig 2B displays Larvae: 0.20 ± 0.34 and Metamorphs: 2.06 ± 0.64. please check them carefully.

Comment 5: Several cross-references, citation years, and terminology should be checked very carefully. Examples include the reference to Table 1 rather than Table 2 for the number of feeding sequences, the reference to Fig. S1 rather than Fig. S3 for the residual diagnostics. Also, I noticed inconsistent years for references. A few examples only include: Gálvez et al., Koumoundouros et al., and Schmidt-Nielsen.

Comment 6: A final language check is still needed. Examples include "cofounding" instead of "confounding," "compensate the loss" instead of "compensate for the loss," inconsistent capitalisation of variable names, and the use of "et" rather than "and" between author names in English prose (Heiss et Grell 2019, Lauder et Shaffer, etc.).

Comment 7: I noted several inconsistencies between the supplementary tables and the main manuscript that should be resolved before publication. Most importantly, Table S2 reports a significant developmental stage × SVL interaction for Mhd in the full model (estimate = −1.15, 95% CI = −2.27 to −0.04, p = 0.043), whereas Results and Methods state that this interaction was not significant and interpret the reduced model. Please reconcile Table S2, Table 1, Fig 2C, and the associated text.

In addition, Table S1 contains 96 larval and 47 metamorphic sequences, whereas the Methods report 96 and 48, respectively. The prey assignments also differ: Table S1 indicates that M1, M2, M3 and M5 received bloodworms and M4 and M6-M10 received earthworms, whereas the Methods assign M1-M4 and M5-M10 to these prey types. Please carefully check, verify and correct throughout.

Reviewer's Responses to Questions

Experimental quality
Are experiments performed using appropriate methods that will answer the question (or test the hypothesis or support the observations) posed by the authors? Is the right tool used for the job?
If 'No', please indicate reasons in Comments for Author box below.
Reviewer #1:

© 2026. Published by The Company of Biologists under the terms of the Creative Commons Attribution License (https://creativecommons.org/licenses/by/4.0/).

- Yes

Reviewer #2:
- Yes

Were the data analyzed using appropriate statistical tests?
If 'No', please indicate reasons in Comments for Author box below.
Reviewer #1:
- Yes

Reviewer #2:
- Yes

Reproducibility
Were experiments performed using adequate number of biological replicates?
If 'No', please indicate reasons in Comments for Author box below.
Reviewer #1:
- Yes

Reviewer #2:
- Yes

Is there sufficient raw data to assess the rigor of the analysis?
If 'No', please indicate reasons in Comments for Author box below.
Reviewer #1:
- No

Reviewer #2:
- Yes

Does the methods section provide sufficient detail to permit reproducibility?
If 'No', please indicate reasons in Comments for Author box below.
Reviewer #1:
- Yes

Reviewer #2:
- Yes

Completeness
Are the manuscript's conclusions supported by the data?
If 'No', please indicate reasons in Comments for Author box below.
Reviewer #1:
- Yes

Reviewer #2:
- No

Scholarship
Do the authors cite and discuss the merits of data that would argue for and against their conclusion?
If 'No', please indicate reasons in Comments for Author box below.
Reviewer #1:
- Yes

© 2026. Published by The Company of Biologists under the terms of the Creative Commons Attribution License (https://creativecommons.org/licenses/by/4.0/).

Reviewer #2:

- Yes

Does the manuscript title & abstract accurately reflect the contents of the manuscript, without hyperbole?
If 'No', please indicate reasons in Comments for Author box below.
Reviewer #1:

- Yes

Reviewer #2:

- Yes

Second revision

Author response to reviewers’ comments

Comments from the Reviewers:

Reviewer 1: I have read the responses to review, and the revised version of the manuscript. I am satisfied with the authors' revisions to this article. The readability and flow have been significantly improved and I feel the results and particularly the discussion, provide novel insights that are of value to the community. There is only one minor issue that remains. The authors declare that videos will be made available on request. The videos are actually the raw datasets for this paper, and as such, would be better made openly available at the time of publication. I would like to ask the authors to upload the videos into an online data repository and to provide the link to the raw video datasets under Data Availability, so that they are accessible to the readers.

OUR REPLY: We thank Reviewer 1 for the positive assessment of the revised manuscript. Although we would be happy to upload the raw videos to an online repository the shear size of the data (over 2Tb) limits our ability to do so. Notably, the estimated cost of depositing the dataset on Dryad would exceed $12,000, which is unfortunately beyond the resources available to us (see Dryad submission cost https://datadryad.org/help/requirements/costs). We have therefore retained the statement in the Data Availability section that the videos are available upon request. We are nevertheless happy to share the data with any researcher interested.

Reviewer 2: I appreciate the author's efforts in addressing my previous comments. The revised manuscript is substantially improved, and the authors have addressed most of my main concerns. In particular, I am pleased to see that the Discussion is now more cautious and now acknowledges some limitations.

I nevertheless think that some issues remain before the manuscript is ready for publication. I am not suggesting any new experiments, but I think that the points below should be addressed prior to the publication. There are so many small inconsistencies in reporting of the values. I could not list all of them, but they are critical and should be fixed.

OUR REPLY: We thank the reviewer for the positive assessment of our revised manuscript, as well as for the careful reading and constructive feedback. We have revised the manuscript accordingly, correcting the identified inconsistencies, language and formatting issues, and have also conducted a thorough proofreading to ensure that similar errors were addressed throughout the text. Moreover, the Discussion now acknowledges the limitations highlighted by the reviewer. Our responses to the specific points are provided below.

Comment 1: Larvae and metamorphs still occupy completely non-overlapping SVL ranges. The models can compare the scaling relationships within each developmental stage, but this does not mean that developmental-stage effects are fully independent of body size. I believe that

© 2026. Published by The Company of Biologists under the terms of the Creative Commons Attribution License (https://creativecommons.org/licenses/by/4.0/).

statements such as metamorphs having greater hyoid depression "independent of body size" should be a little bit softened. The lack of size overlap should also be acknowledged as a limitation of the stage comparisons, rather than only in relation to maximum gape.

OUR REPLY: We have softened the statement regarding the effect of developmental stage and now state that "hyoid depression is mainly impacted by developmental stage." In addition, we now explicitly acknowledge the lack of overlap in SVL between larvae and metamorphs as a limitation of the stage comparisons in the Discussion (L340-348)

Comment 2: The revised manuscript now acknowledges that larvae and some metamorphs were fed bloodworms, whereas other metamorphs were fed pieces of earthworm. However, the inspection of the raw patterns cannot exclude the possible effect of prey size, type, or stiffness on the observed kinematics. A sensitivity analysis would be useful (provided that the remaining sample is adequate?). Otherwise, the authors would need to avoid suggesting that prey effects have been ruled out or should add commentaries.

OUR REPLY: We now explicitly acknowledge that differences in prey size and/or type among some metamorphs could potentially have influenced the observed kinematics when discussing the buccal expansion variables (L201-202) and the duration variables (L223-224). For the duration variables, however, we also discuss why we consider a prey-size effect unlikely to explain the observed pattern (L224-228). Specifically, if larger prey were responsible for the differences observed in metamorphs, we would expect the increased prey size to increase movement duration rather than decrease it.

Comment 3: The captions of Figs 3-5 incorrectly state that a significant slope has a 95% confidence interval "above zero." I can see that some significant slopes are negative. Therefore, this should be changed to "the 95% confidence interval excludes zero."

OUR REPLY: We have corrected the captions of Figures 3-5.

Comment 4: The numerical values reported in the text should be checked very carefully against those shown in the figures. For example, the MGA slope values and the reported DhdE slope ratio appear to be inconsistent across different parts of the manuscript. I could not list all of them but an example is where Discussion reports slopes of 0.28 for larvae and 2.28 for metamorphs, but Fig 2B displays Larvae: 0.20 ± 0.34 and Metamorphs: 2.06 ± 0.64. please check them carefully.

OUR REPLY: The inconsistencies mentioned arose because some numerical values in the text had not been updated following the latest revision of our statistical analyses. We have now carefully cross-checked all numerical values reported in the manuscript against those presented in the figures and tables and have corrected the discrepancies.

Comment 5: Several cross-references, citation years, and terminology should be checked very carefully. Examples include the reference to Table 1 rather than Table 2 for the number of feeding sequences, the reference to Fig. S1 rather than Fig. S3 for the residual diagnostics. Also, I noticed inconsistent years for references. A few examples only include: Gálvez et al., Koumoundouros et al., and Schmidt-Nielsen.

OUR REPLY: We have corrected these discrepancies.

Comment 6: A final language check is still needed. Examples include "cofounding" instead of confounding," "compensate the loss" instead of "compensate for the loss," inconsistent capitalization of variable names, and the use of "et" rather than "and" between author names in English prose (Heiss et Grell 2019, Lauder et Shaffer, etc.).

OUR REPLY: We have corrected the identified errors and thoroughly checked the rest of the manuscript.

Comment 7: I noted several inconsistencies between the supplementary tables and the main manuscript that should be resolved before publication. Most importantly, Table S2 reports a significant developmental stage × SVL interaction for Mhd in the full model (estimate = −1.15, 95%

© 2026. Published by The Company of Biologists under the terms of the Creative Commons Attribution License (https://creativecommons.org/licenses/by/4.0/).

CI = −2.27 to −0.04, $p = 0.043$), whereas Results and Methods state that this interaction was not significant and interpret the reduced model. Please reconcile Table S2, Table 1, Fig 2C, and the associated text.

OUR REPLY: We thank the reviewer for bringing these inconsistencies to our attention. They were due to an oversight when implementing the sjPlot function. While our model estimates and p-values were initially obtained using the summary function with the Satterthwaite method, we used the sjPlot function to generate the Table S2 output but inadvertently forgot to specify the Satterthwaite method. As a result, the p-values in the table were calculated using a different method. This has been corrected in the current version of the script and Table S2.

In addition, Table S1 contains 96 larval and 47 metamorphic sequences, whereas the Methods report 96 and 48, respectively. The prey assignments also differ: Table S1 indicates that M1, M2, M3 and M5 received bloodworms and M4 and M6-M10 received earthworms, whereas the Methods assign M1-M4 and M5-M10 to these prey types. Please carefully check, verify and correct throughout.

OUR REPLY: We have corrected these inconsistencies throughout the manuscript.

Third decision letter

MS ID#: bio.062752R2

MS Title: The impact of metamorphosis and size on the aquatic feeding kinematics of the Iberian ribbed newt (Pleurodeles waltl Michahelles, 1830)

Authors: Isabelle Toussaint-Lardé; Merlin Hugon-Daune-Fäs; Anthony Herrel; Anne-Claire Fabre

I am happy to tell you that your manuscript has been accepted for publication in Biology Open, pending our standard publication integrity checks. It was accepted on 28th August 2026.

© 2026. Published by The Company of Biologists under the terms of the Creative Commons Attribution License (https://creativecommons.org/licenses/by/4.0/).
